# β2 Integrin CD11d/CD18: From Expression to an Emerging Role in Staged Leukocyte Migration

**DOI:** 10.3389/fimmu.2021.775447

**Published:** 2021-11-08

**Authors:** Eoin N. Blythe, Lynne C. Weaver, Arthur Brown, Gregory A. Dekaban

**Affiliations:** ^1^Molecular Medicine Research Laboratories, Robarts Research Institute, University of Western Ontario, London, ON, Canada; ^2^Department of Microbiology and Immunology, University of Western Ontario, London, ON, Canada; ^3^Department of Physiology and Pharmacology, University of Western Ontario, London, ON, Canada; ^4^Department of Anatomy and Cell Biology, University of Western Ontario, London, ON, Canada

**Keywords:** beta 2 integrin, CD11d, CD18, leukocyte, migration, extravasation, inflammation

## Abstract

CD11d/CD18 is the most recently discovered and least understood β2 integrin. Known CD11d adhesive mechanisms contribute to both extravasation and mesenchymal migration – two key aspects for localizing peripheral leukocytes to sites of inflammation. Differential expression of CD11d induces differences in monocyte/macrophage mesenchymal migration including impacts on macrophage sub-set migration. The participation of CD11d/CD18 in leukocyte localization during atherosclerosis and following neurotrauma has sparked interest in the development of CD11d-targeted therapeutic agents. Whereas the adhesive properties of CD11d have undergone investigation, the signalling pathways induced by ligand binding remain largely undefined. Underlining each adhesive and signalling function, CD11d is under unique transcriptional control and expressed on a sub-set of predominately tissue-differentiated innate leukocytes. The following review is the first to capture the nearly three decades of CD11d research and discusses the emerging role of CD11d in leukocyte migration and retention during the progression of a staged immune response.

## Introduction

The four members of the β2 integrin family, which are surface expressed only on leukocytes, have critical functions within the innate and adaptive immune systems (1, 2). Various β2 integrin nomenclatures exist, resulting in each member having multiple designations: CD11a/CD18 (α_L_β_2_, LFA-1, alphaLbeta2), CD11b/CD18 (α_M_β_2_, Mac-1, CR3, alphaMbeta2), CD11c/CD18 (α_X_β_2_, p150.95, CR4, alphaXbeta2) and CD11d/CD18 (α_D_β_2_, alphaDbeta2) (1, 2). For the following review, the CD11/CD18 nomenclature will be used exclusively. Acting as adhesion receptors, β2 integrins induce leukocyte adhesion and transmit bidirectional signals (1). Inside-out signalling describes signalling molecules binding to the cytoplasmic tail and inducing a change in integrin conformation. Outside-in signalling describes ligand binding to the extracellular I domain and transmission of a signal into the cytoplasm (3). The collection of adhesive and signalling mechanisms form the functionality of β2 integrins in leukocyte trafficking, cytokine release, phagocytosis, toll like receptor (TLR) signalling, B cell receptor (BCR) signalling, immunological synapse signalling, and targeted cell killing (1).

CD11d/CD18 is an understudied member of the β2 integrin family. First characterized in canines in 1995 (4), CD11d is now understood to be expressed by a variety of human leukocytes (5, 6) and to have both adhesion and signalling functions (7, 8). This review aims to examine known CD11d structure, expression, functionality, associated pathophysiological states, and targeted immunomodulatory agents. A focus of CD11d study has been its role in leukocyte migration, retention, and its contribution to the harmful accumulation of leukocytes in various pathophysiological states. Currently, two separate groups are developing CD11d-targeted agents to modulate the harmful recruitment of leukocytes following acute neurotrauma (9–12) and during chronic inflammatory disease (13). Less is known regarding the CD11d protein structure and bidirectional signalling pathways that have been determined for the other known β2 integrins. Sequence comparisons, predicted structures, and predicted functionalities will be presented alongside the known β2 integrin counterparts to analyse these lesser-known aspects of CD11d biology.

## General β2 Integrin Structure

**Figure 1:**
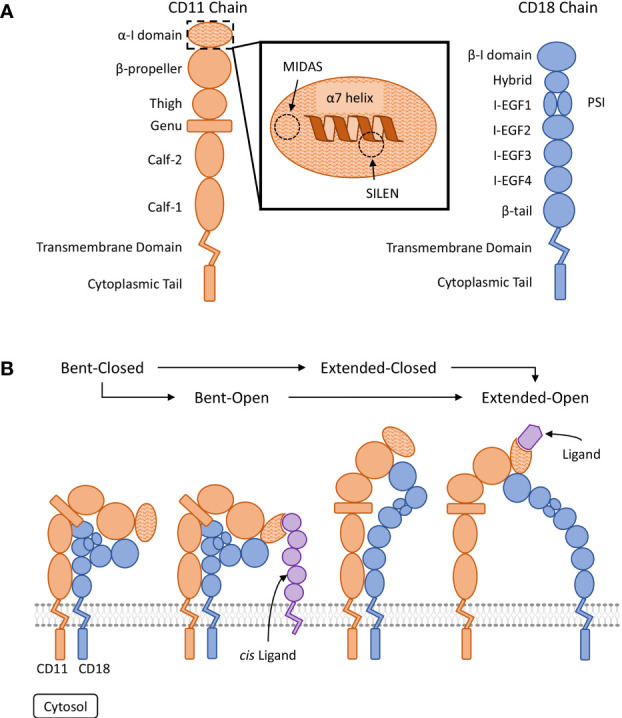
Visual representation of β2 integrin structure and conserved regulatory conformations. **(A)** Organization of the domains composing the CD11 and CD18 chains (15, 16). The ligand binding α-I domain is highlighted by a hatched pattern. The metal ion-dependant adhesion site (MIDAS) and the socket for isoleucine (SILEN) motifs are located within the α-I domain (16, 17). The SILEN motif interacts with an invariant isoleucine located in the α7 helix to maintain the inactive conformation (17). **(B)** Representation of the β2 integrin regulatory conformations. The bent-closed inactive conformation predominates under basal conditions (1). Stimulation can activate the integrin and induce the extended-closed conformation. Additional stimulation and the binding of a divalent metal ion to the MIDAS motif, can induce the extended-open conformation. The bent-open conformation is stabilized by binding a *cis* ligand and may provide an alternative activation pathway to the extended-open conformation (18–20). The extended-open conformation is characterized by the separation of the cytosolic tails in additional to local conformational changes within the α-I domain, including shifting of the α7 helix (14–16, 21, 22).

Each β2 integrin is a heterodimeric type I transmembrane protein composed of a variable alpha chain (CD11a-d) and a common beta chain (CD18). The two chains dimerize non-covalently and each consists of several extracellular domains, a singular transmembrane domain, and a short cytoplasmic tail (14) (**Figure 1**). The conformation and clustering of integrins heavily regulate their ligand avidity and functionality on the cellular membrane (21, 22). Multiple factors impact integrin conformation and clustering including inside-out signalling, outside-in signalling and thermodynamic equilibriums (21, 23, 24). Four conserved conformations have been observed across CD11a-c that are labelled: bent-closed, bent-open, extended-closed, and extended-open (**Figure 1**) (15, 16, 21, 22, 25, 26). The bent-closed conformation has a low ligand affinity and is regarded as inactive. Stimulatory signals can activate the integrin resulting in the extension of the extracellular domains into an extended-closed conformation before the transition to the fully activated extended-open conformation. The fourth conformation, bent-open, may allow for an alternative transition pathway to the extended-open conformation (25, 26). The bent-open conformation is stabilized by the binding of *cis* ligands – a ligand that is present on the surface of the β2 integrin expressing leukocyte (18–20). The detailed processes involved in integrin activation are beyond the scope of this review and we refer the reader to the following articles (16, 21, 27). In leukocytes under basal conditions, β2 integrins predominately favour an inactive bent-closed conformation that binds ligands with low affinity (1, 16).

## CD11d Genetics and Transcription Factors

The gene encoding CD11d, *ITGAD*, is located downstream of the *ITGAM* (CD11b) and *ITGAX* (CD11c) genes on chromosome 16 (28–30). All three genes are encoded in the same direction and clustered separately from *ITGAL* (CD11a), which is also located on chromosome 16 (31). Phylogenetic analysis echoes these observations with CD11a diverging from a common CD11b-d ancestor (32). The encoded CD11d amino acid sequence shares the greatest homology with CD11c (70%), followed by CD11b (59%), while much less homology is shared with CD11a (32%) (33). Each known CD11 chain has a short and long isoform due to alternative splicing (28–31). The CD11d short isoform differs from the long isoform by the absence of a glutamine at residue 500 (30). No study has directly investigated the potential differences between these two CD11d isoforms. Inclusion and exclusion of the signal peptide in the residue numbering of the CD11 chain varies between separate studies. For consistency this review will position residues along the respective long CD11 isoform and numbering will include the signal peptide sequence.

Mouse models are commonly used to study CD11d because of the presence of a murine ortholog to each known β2 integrin. The positioning of all four murine β2 integrin orthologs echoes the pattern observed in humans. Murine *Itgad*, *Itgax*, and *Itgam* are encoded in the same direction and clustered separately from *Itgal* on chromosome 7 (34–37). Targeted deletion has created CD11d^-/-^ mice that lack a functional copy of the *Itgad* gene. CD11d^-/-^ mice display normal growth, development, fertility, IgG serum levels, peripheral leukocytes counts, and no increase in spontaneous infection (38, 39). The weight of murine CD11d^-/-^ spleens was noted to be heavier than wildtype spleens at weeks 10-11 but no difference was recorded at weeks 17-18 (38).

The human *ITGAD* gene is under unique transcriptional regulation compared to the other β2 integrins (40, 41). Sp1 and Sp3 are shared transcription factors involved in the regulation of the CD11a-d and CD18 promoters (40). Sp1 is expressed equally across all leukocytes and induces the basal expression of CD11d. Meanwhile, Sp3 alters Sp1 basal expression with cell-type specific repressor and activator functions (42). Divergent to CD11a-c, transforming growth factor-β-inducible early gene-1 (TIEG1) and two isoforms of gut-enriched Kruppel-like factor 4 (GKLF/GKLFa) interplay with Sp1 to regulate CD11d in a cell and stimulant-specific manner (40, 41). TIEG1, GKLF/GKLFa, and Sp1 bind to the CD11d promoter at a common binding site *via* zinc-finger DNA binding domains (40). TIEG1 contains three repression domains and represses CD11d expression in non-myeloid cells; however, in differentiated myeloid cells an increase in TIEG1 binding is coupled with CD11d upregulation (41). The reported role of TIEG1 in CD11d activation is novel and the exact mechanism of cell-specific CD11d activation remains unclear. A leading hypothesis states that the competition or interaction between TIEG1 and a set of transcription factors may sum to form the observed activation of CD11d in differentiated myeloid cells (41). The complete set of CD11d transcription factors responsible for these cell-specific responses is unknown. One known transcription factor that does compete with TIEG1 to bind the CD11d promoter and likely impacts CD11d expression is GKLF/GKLFa. Histone deacetylase 1 and 2 associate with GKLF/GKLFa bound to the CD11d promoter to repress CD11d expression across myeloid, non-myeloid, and differentiated myeloid cells (40). Sp1, TIEG1, and GKLF/GKLFa all contribute to the bimodal CD11d response to phorbol myristate acetate (PMA) stimulation observed in myeloid cell lines. Acute 24-hour 10nM PMA stimulation decreases Sp1 binding, maintains GKLF/GKLFa binding, and decreases CD11d mRNA expression. Prolonged 48-hour 100nM PMA stimulation, representing myeloid differentiation, corresponds with the release of GKLF/GKLFa, resurgence of Sp1, increase in TIEG1, and CD11d mRNA upregulation (40, 41).

A putative transcription factor also involved in CD11d regulation is proliferator-activated receptor-γ (PPAR-γ). Mice deficient in PPAR-γ have splenic red pulp macrophages with substantially downregulated CD11d mRNA while CD11a and CD11b mRNA are upregulated (43). Furthermore, sequence analysis identifies several potential binding sites for PPAR-γ within the CD11d promoter (43). PPAR-γ is a member of the nuclear hormone receptor superfamily and is expressed in a range of leukocytes including monocytes/macrophages, neutrophils, lymphocytes, and dendritic cells (44). The confirmation of PPAR-γ binding to the CD11d promoter and its direct impacts on CD11d regulation have yet to be reported. A separate study has also postulated a set of CD11d transcription factors that are involved in myeloid cell differentiation. Oxidized and acetylated low-density lipoproteins (Ox-LDL and Ac-LDL) induce the upregulation of CD11d mRNA during HL60 foam cell formation, but do not impact CD11d mRNA expression in foam cells (45). Foamy macrophages are known to be involved in atherosclerosis and drive plaque formation (46). Additionally, chronic spinal cord injury pathophysiology presents foamy macrophages within the injury lesion (47). A potential candidate for this unknown transcription factor involved in foam cell formation and CD11d expression is PPAR-γ (43). PPAR-γ binds to Ox-LDL, is expressed within monocytes/macrophages, and is involved in foam cell formation (44, 48, 49). Further investigation is warranted to characterize PPAR-γ as a putative CD11d transcription factor and its possible connection to CD11d upregulation in response to Ox-LDL during foam cell formation.

## CD11d Structure and Post-Translational Modifications

**Figure 2:**
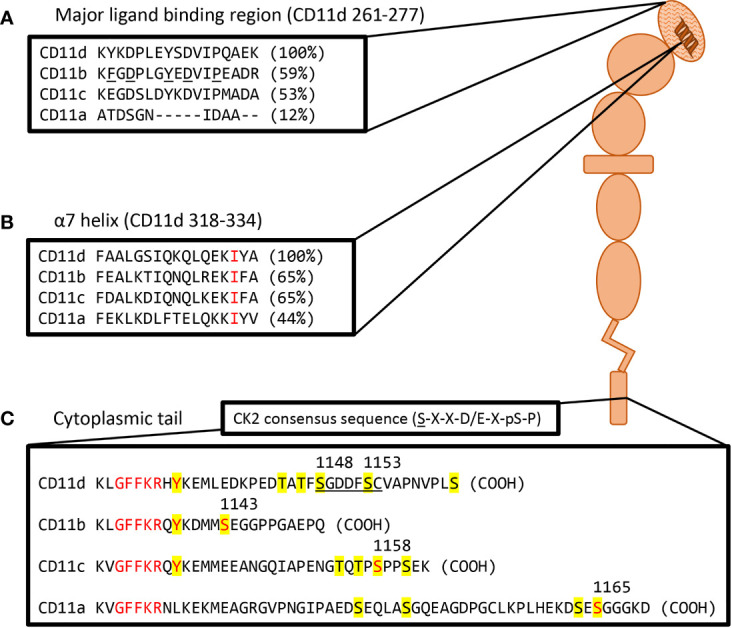
Visual representation of a probable CD11d structure including amino acid homolog comparisons of key motifs. **(A)** Sequence comparison of the CD11d α-I domain major ligand binding region. The ligand binding CD11d α-I domain is highlighted by a hatched pattern. Residues determined to be important in the ligand binding pocket of CD11b are underlined and percent homology to CD11d is in brackets. Alignment and CD11b residue analysis performed in previous study (51). **(B)** Sequence comparison of the CD11 α7 helix. An invariable isoleucine is highlighted in red and percent homology to CD11d is in brackets. Alignment was performed in previous study (17). Conformational changes to the α7 helix within CD11d have been shown to alter ligand affinities, thus implying the presence of an open and closed α-I domain conformation (51). **(C)** Sequence comparison of complete CD11 cytoplasmic tails. Yellow denotes potential phosphorylation sites, red denotes conserved residues of interest, and the underlined sequence denotes a potential CK2 site. The GFFKR “hinge” motif is required to maintain the association of the CD11 and CD18 cytoplasmic tails (52). The constitutive phosphorylation of a serine residue is conserved across CD11a (Ser^1165^) (53), CD11b (Ser^1143^) (54), and CD11c (Ser^1158^) (55). CD11d has a putative CK2 site at Ser^1148^-Cys^1154^ using the consensus sequence (S-X-X-D/E-X-pS-P) (56). The same sequence would predict Ser^1153^ to be constitutively phosphorylated as observed in other β2 integrins. The function of the conserved GFFKR +2 tyrosine residue in CD11b-d is largely undefined. The tyrosine appears to be embedded into the membrane during the inactive conformation, while exposed during the active conformation (57). Long isoform CD11a (NP_002200.2), CD11b (NP_001139280.1), CD11c (NP_000878.2), and CD11d (NP_001305114.1) amino acid sequences were acquired from the National Center for Biotechnology Information database (28–31).

Certain conserved motifs heavily regulate the tertiary structure of the CD11 chain and mediate the known conformational changes. The open and closed integrin conformations refer to the state of the α-I domain regulated by a metal ion-dependant adhesion site (MIDAS) and socket for isoleucine (SILEN) (50) (**Figure 1**). These two motifs can impact the position of the α7 helix to either stabilize the closed or open α-I domain state. Under basal conditions, the SILEN motif acts to stabilize the closed α-I domain conformation by interacting with an invariable isoleucine within the CD11 α7 helix (17) (**Figure 2**). Activation can shift the position of the α7 helix and open the α-I domain MIDAS motif for divalent metal ion binding (14, 16, 17). It is important to note that not all divalent metal ions have the same effect on the α-I domain conformation. In CD11b, Mg^2+^ binds the MIDAS motif to stabilize the open state, while Ca^2+^ binds the MIDAS motif to stabilize the closed state (58). Separation of the CD11 and CD18 cytosolic tails is another key conformational change during integrin activation. The conserved GFFKR or “hinge” motif maintains the association of the cytosolic tails during the inactive state (**Figure 2**). Deletions within the GFFKR sequence activates the integrin to a high affinity state (52). Interestingly, the conserved GFFKR +2 tyrosine in CD11b-d is postulated to be hidden within the membrane in the inactive conformation, while accessible for potential phosphorylation in the active conformation (57). The role of the conserved GFFKR +2 tyrosine has yet to be defined but it may play a part in outside-in signalling. Regarding the presence of these motifs within CD11d, a crystallized structure has yet to be elucidated thus the conservation of these structures within CD11d have not been confirmed. Conservation of key sequences, however, predict the existence of similar structures within CD11d (**Figure 2**). Alterations to the predicted CD11d α7 helix sequence can induce a constitutively active or inactive affinity state, thus supporting the conservation of the open and closed α-I domain conformations (51). Ultimately, structural studies are still required to confirm the presence of the bent-closed, bent-open, extended-closed, and extended-open conformations in CD11d/CD18.

Phosphorylation of the CD11 and CD18 cytoplasmic tails are key to the signalling mechanisms of β2 integrins. The cytoplasmic tails of CD11a-c are found to be constitutively phosphorylated whereas the cytoplasmic tail of CD18 is phosphorylated upon activation (59, 60). Notably in CD11a-c, a constitutively phosphorylated serine residue on the cytoplasmic tail is a required step for complete integrin activation (**Figure 2**). Deletion of these serine phosphorylation sites prevents complete activation and decreases ligand affinity (53–55). On the CD18 chain, phosphorylation of Thr^758^ during inside-out activation is a subsequent requirement for complete integrin activation. Deletion of the CD18 Thr^758^ phosphorylation site impairs adhesion and actin mobilization (61). No study has yet confirmed a homologous CD11d constitutively phosphorylated serine; however, three serine residues do exist in the CD11d cytoplasmic tail, as well as a putative CK2 phosphorylation site not observed in the other β2 integrins. The constructed CK2 consensus sequence (56) – S-X-X-D/E-X-pS-P – predicts CK2 phosphorylation at position S given prior phosphorylation at position pS. Thus, the putative CD11d CK2 phosphorylation site at Ser^1148^-Cys^1154^ predicts phosphorylation at Ser^1148^ given constitutive phosphorylation at Ser^1153^ (**Figure 2**). The importance of phosphorylating the alpha chain during outside-in signalling differs between integrin families. Alpha chain phosphorylation is involved with the respective outside-in signalling pathways of integrins α3Aβ1, α6Aβ1, and α6Aβ4 (62). In comparison, the involvement of alpha chain phosphorylation in β2 integrin outside-in signalling has yet to be demonstrated (62, 63). The unique presence of a putative CD11d CK2 phosphorylation site may translate to the involvement of alpha chain phosphorylation in CD11d/CD18 outside-in signalling.

## CD11d Ligand Specificity

**Figure 3:**
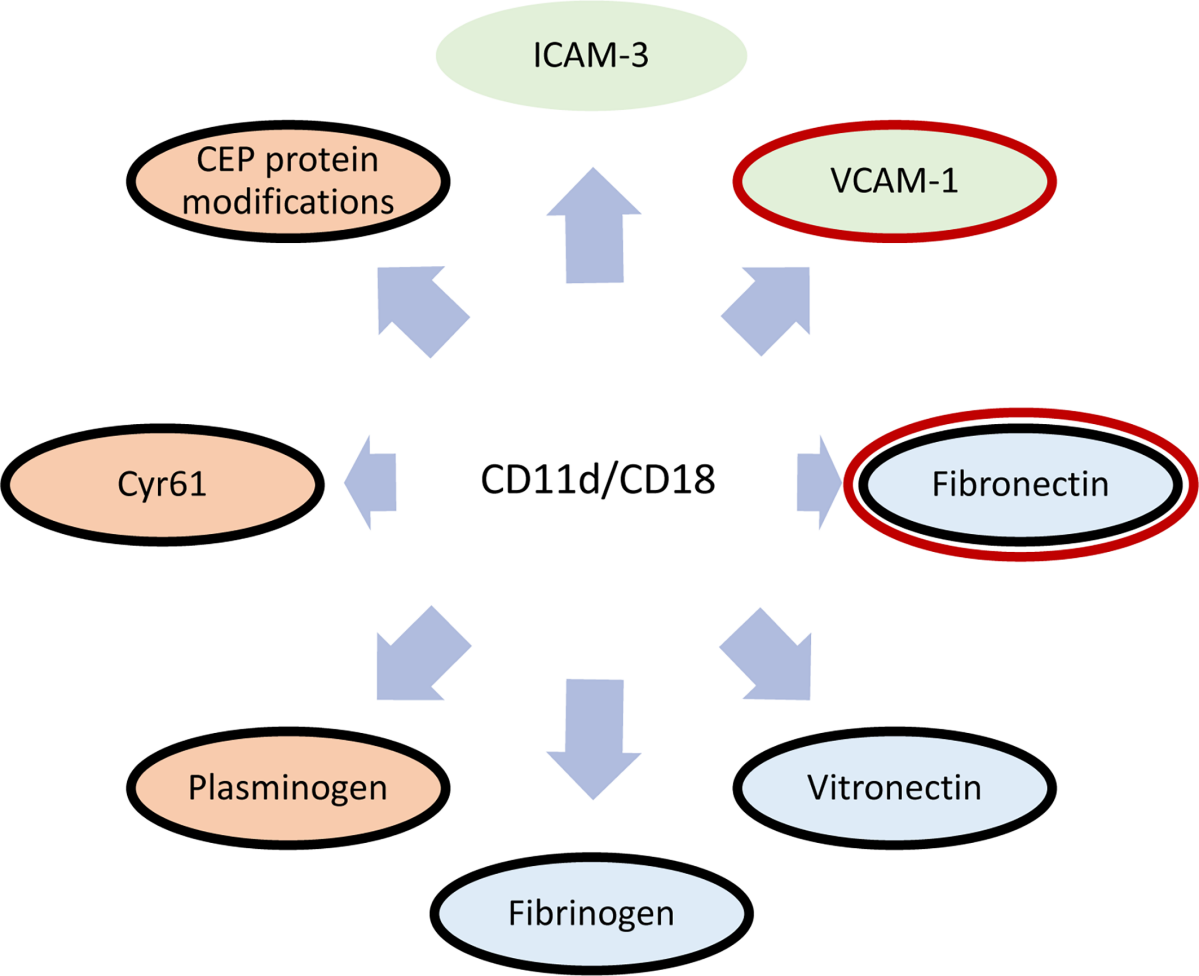
Diagram of known CD11d/CD18 ligands. Cellular receptors are shaded green, extracellular matrix proteins are blue, and proteins/protein modifications prevalent within the ECM during inflammation are red (51, 68). Shared ligand specificities with CD49d/CD29 is denoted in a red outline (70), while shared ligand specificities with CD11b/CD18 is outlined in black (51).

The functions of CD11d/CD18 heavily revolve around the CD11d ligand binding specificity. β2 integrins are widely known to bind ligands *via* the α-I domain (64–66). Initial studies demonstrated the binding of human CD11d/CD18 to both human vascular cell adhesion molecule-1 (VCAM-1) and induced endothelial cell adhesion molecule-3 (ICAM-3) *via* the α-I domain (5, 67, 68). The binding affinity for VCAM-1 was found to be greater than that of ICAM-3 (68). Later work identified promiscuous binding to extracellular matrix (ECM) associated proteins including; fibrinogen, vitronectin, fibronectin, Cyr61, and plasminogen (51). Recently, CD11d has been described to bind the protein modification 2-(ω-carboxyethyl)-pyrrole (CEP), which is a by-product of lipid peroxidation (13, 69) (**Figure 3**). As a group, bent-open β2 integrins have been shown to bind sialylated FcγRIIA, ICAM-1, and ICAM-3 expressed on the same leukocyte (18–20). While CD11d has been shown to bind ICAM-3, there has yet to be direct evidence of CD11d binding ICAM-3 in a bent-open conformation.

Residues involved in the CD11d ligand binding site were first discovered through homolog studies with CD11b (51). Structural studies of CD11b determined that Lys^261^-Arg^277^ diverges from CD11a, bestowing promiscuous ligand binding to the CD11b α-I domain. CD11d shares 60% amino acid sequence homology with the α-I domain of CD11b and a similar Lys^261^-Lys^277^ sequence that is also important for promiscuous ligand binding (**Figure 2**). A CD11a/CD18 chimera containing the CD11d Lys^261^-Lys^277^ sequence shares the CD11d/CD18 ligand binding specificity but with unique binding affinities (51). Additional residues, therefore, contribute to the complete CD11d binding site. Future structural studies are required to determine the structure of this CD11d ligand binding site and determine key residues.

## CD11d Expression

The understanding of human CD11d expression has evolved over time with expression reported in select human myeloid and lymphoid cells. Initial investigations noted low expression of CD11d amongst peripheral blood leukocytes, moderate expression on myeloid cells, and strong expression on tissue-specialized myeloid cells including splenic red pulp macrophages and granulocytes, synovial macrophages, and foamy macrophages (5, 71). Key exceptions were moderate CD11d expression on peripheral eosinophils and an absence of CD11d on liver specialized Kupffer cells (5, 67). A later study further investigated the expression of CD11d on lymphoid cells and revealed strong expression on B cells and NK cells. Amongst T cells, γδ T cells express CD11d at consistently greater levels than αβ T cells, while Vδ1 surface expression on CD11d^+^ γδ T cells is more prevalent than Vδ2 (6) (**Table 1**).

**Table 1:** Basal CD11d expression amongst leukocytes of various species.

Organism	Tissue	Cell Type or Sample Source	Basal Expression	Reference
Human	Periphery	CD14^++^CD16^-^ monocyte	+	(7, 72)^AB,A^
		CD14^+^CD16^+^ monocyte	+	(7, 72)^AB,A^
		B cell	+	(6)^A^
		NK cell	++	(6, 73)^A,B^
		αβ T cell	-/+	(6)^A^
		γδ T cell	+	(6)^A^
		Eosinophil	+	(67)^A^
		Basophils	++	(67)^A^
		Neutrophil	+	(7, 67, 73, 74)^A,A,A,B^
	*In vitro* cultured	Myeloid derived dendritic cell	+	(7)^B^
		Myeloid derived monocyte	+	(7)^B^
		6-sulfo LacNAc^+^ dendritic cell	++	(73)^B^
	Spleen	Splenic red pulp macrophage	++++	(5)^C^
		Splenic red pulp granulocyte	++++	(5)^C^
	Synovial Joints	Synovial macrophage	++	(71)^C^
	Liver	Kupffer cell	–	(5)^C^
Mouse	Periphery	Peripheral leukocyte	-/+	(38, 39, 75)^A,A,B^
		Neutrophil	+	(76)^A^
		Monocyte	+	(76)^A^
	Spleen	Splenic red pulp macrophage	++++	(38, 77)^BC,D^
	Peritoneum	Peritoneal macrophage	-/+	(38, 78)^A,B^
	Bone Marrow	Macrophage	++	(38)^BC^
	Thymus	Macrophage	++	(38)^BC^
	Liver	Kupffer cell	–	(38)^B^
	Lungs	Lung homogenate	-/+	(77)^D^
Rat	Periphery	Peripheral leukocyte	-/+	(79)^A^
		Neutrophil	-/+	(79)^AB^
		Monocyte	-/+	(79)^AB^
	Lungs	Lung homogenate	+	(80)^E^
	Spleen	Splenic red pulp macrophage	++++	(79)^AE^
Canine	Periphery	Peripheral leukocyte	-/+	(4)^A^
		CD8^+^ T cell	+	(4)^A^
	Spleen	Splenic red pulp macrophage	++++	(4)^AC^
	Liver	Kupffer cell	–	(4)^AC^

Expression is represented on a scale of – (not detected) to ++++ (highly expressed). A limitation encountered when comparing CD11d expression across studies was the variation in detection method: flow cytometry (A), immunocytochemistry (B), immunohistochemistry (C), quantitative PCR (D), and western blot (E).

Detection of CD11d expression does vary across species. Under basal conditions, CD11d expression is detected at low levels on murine peripheral blood leukocytes including neutrophils and monocytes (38, 76). One study has described that murine T cells lack CD11d surface expression, while a separate study has reported surface CD11d expression on γδ T cells and αβ T cells (39, 75). Both studies used flow cytometry. In canines, CD11d surface expression is extremely low on peripheral leukocytes, but is present on a small portion of CD8^+^ T cells (4). Tissue-specialized myeloid cells mainly conserve the pattern of expression across observed species. CD11d protein expression is consistently detected in human, canine, and mouse splenic red pulp macrophages; however, CD11d is consistently absent from liver Kupffer cells (4, 5, 38). IC-21 cells – a peritoneal macrophage cell line from C57BL/6 mice – express CD11d and have been used to model CD11d ligand binding interactions (51). Finally, investigation within a rat model demonstrated low CD11d surface expression amongst peripheral leukocytes and consistent expression amongst splenic macrophages (79). Rat alveolar macrophages also express low levels of CD11d protein (80) (**Table 1**).

Regulation of CD11d expression is cell-type specific and influenced by the temporal duration of leukocyte stimulation. These regulatory nuances have been modelled across a diverse set of cell lines: THP-1 (monocytic), HL60 (promyelocytic), IM-9 (B-cell), and Jurkat (T-cell) (40–42). In myeloid cell lines (THP-1 and HL60), acute 24-hour 10nM PMA exposure decreases CD11d mRNA, while prolonged 48-hour 100nM PMA exposure substantially increases CD11d mRNA (40, 41, 45). Further analysis is still required to determine if the observed increase in CD11d mRNA results in an increased CD11d/CD18 surface expression. In non-myeloid cells (IM-9 and Jurkat), northern blot analysis did not detect CD11d mRNA before or after PMA stimulation (41, 42). Progressing beyond cell lines, investigations of CD11d upregulation in various pathophysiological states have further characterized the regulation of this β2 integrin. CD11d upregulation has been observed in spinal cord injury (SCI) (74), atherosclerosis (78), obesity (81), arthritis (71), acute lung injury, and acute respiratory distress syndrome (ARDS) patients (7). In peripheral blood eosinophils isolated from the bronchi of allergic patients challenged with allergen, interleukin 5 was found to upregulate CD11d surface expression directly (67) (**Table 2**). An underlying constraint to all CD11d surface level expression is the co-expression with the CD18 β chain. Without the presence of CD18, CD11d is retained in the trans-Golgi network and is not functionally expressed on the cell surface (82).

**Table 2:** Upregulation of CD11d expression during various disease and injury states.

Disease/Injury	Organism	Tissue	Cell Type or Sample Source	Expression	Reference
Atherosclerosis	Human	Atherosclerotic lesion	Foamy macrophage	++++	(5)^C^
	Mouse		Macrophage	++++	(78)^A^
Acute Lung Injury or ARDS	Human	Lungs	Alveolar Macrophage	+++	(7)^C^
	Rats	Lungs	Lung homogenate	+++	(80)^CE^
	Mouse	Lungs	Lung homogenate	+++	(77)^D^
Arthritis	Human	Synovial Joints	Synovial Macrophage	+++	(71)^C^
Allergen Challenged	Human	Lungs	Bronchoalveolar eosinophil	++	(67)^A^
LPS-induced endotoxemia	Mouse	Periphery	Neutrophil	++	(76)^A^
Neurotrauma	Human	Periphery	Neutrophil	+++	(74)^A^
			Monocyte	+++	(74)^A^
Obesity	Human	Subcutaneous WAT	Macrophage	+++	(81)^D^
	Mouse	Retroperitoneal WAT	Macrophage	+++	(81)^D^

Expression is represented on a scale of – (not detected) to ++++ (highly expressed). A limitation encountered when comparing CD11d expression across studies was the variation in detection method: flow cytometry (A), immunocytochemistry (B), immunohistochemistry (C), quantitative PCR (D), and western blot (E).

## CD11d Impact on Leukocyte Migration

### Extravasation

Integrins are known to play an important role in leukocyte extravasation from the periphery into inflamed tissues (83). Leukocyte integrins can interact with endothelium VCAMs and ICAMs to lock the leukocyte onto the endothelium and permit diapedesis (83, 84). The loss of functional β2 integrins impedes leukocyte migration as highlighted in genetic leukocyte adhesion deficiencies (LAD) I and III. In LAD I, CD18 expression is severely diminished, while in LAD III kindlin-3 deficiency prevents the activation of β2 integrins in response to chemoattractants. Both LAD I and III are characterized by impaired leukocyte localization into inflamed tissues and recurrent infections (15). CD11d has been shown to bind VCAM-1 and adhere under sheer flow conditions, thus demonstrating the ability to support leukocyte arrest during extravasation (68). Integrin CD49d/CD29 (very late antigen 4, α4β1), also binds VCAM-1 and has as well established role in leukocyte extravasation (70). Both CD11d and CD49d target overlapping binding sites on VCAM-1 and could potentially have redundant functions during leukocyte extravasation (68). The relative expression of CD11d/CD18 and CD49d/CD29 may dictate their relative contributions to leukocyte extravasation. Under basal conditions, CD11d/CD18 would most likely play a minimal role in VCAM-1 mediated extravasation compared to CD49d/CD29 as peripheral leukocytes express low levels of CD11d. Alternatively, these two integrins could be involved in different stages of extravasation during the progression of an inflammatory response.

Pathology and injury can significantly increase CD11d expression amongst peripheral leukocytes and thus increase their role in extravasation. The relative contribution of CD11d/CD18 to leukocyte extravasation during pathology is difficult to determine because of a shared VCAM-1 binding specificity with CD49d/CD29 (**Figure 3**). A thioglycollate peritonitis model demonstrated no change in extravasation capacity between CD11d^-/-^ and wildtype monocytes (78). It was hypothesized that CD11d/CD18 functions redundantly to CD49d/CD29 during peritoneal extravasation and the loss of CD11d/CD18 was negated by maintained CD49d/CD29 expression (78). In comparison, intravenous treatment with a CD11d-targeted antibody following compression spinal cord injury, but not in the presence of intraspinal haemorrhage, can reduce the infiltration of peripheral myeloid cells (79, 85, 86). These results suggest a non-redundant functionality of CD11d/CD18 during the extravasation of peripheral leukocytes into the injured CNS. Resolving these contradictions may require further exploration of the differences between various CD11d *in vivo* models. First, the contribution of CD11d/CD18 to leukocyte extravasation could logically be linked to its surface density. Neutrophil and monocyte surface expression of CD11d is increased following neurotrauma (74), whereas the unstimulated monocytes used in the thioglycollate peritonitis model express low levels of CD11d (78). Additionally, the differences in physiology may also lead to the described discrepancy. The process of extravasation into the peritoneum has been demonstrated to differ uniquely from other tissues including the lung, skin and cremaster (87).

### Tissue Migration

**Figure 4:**
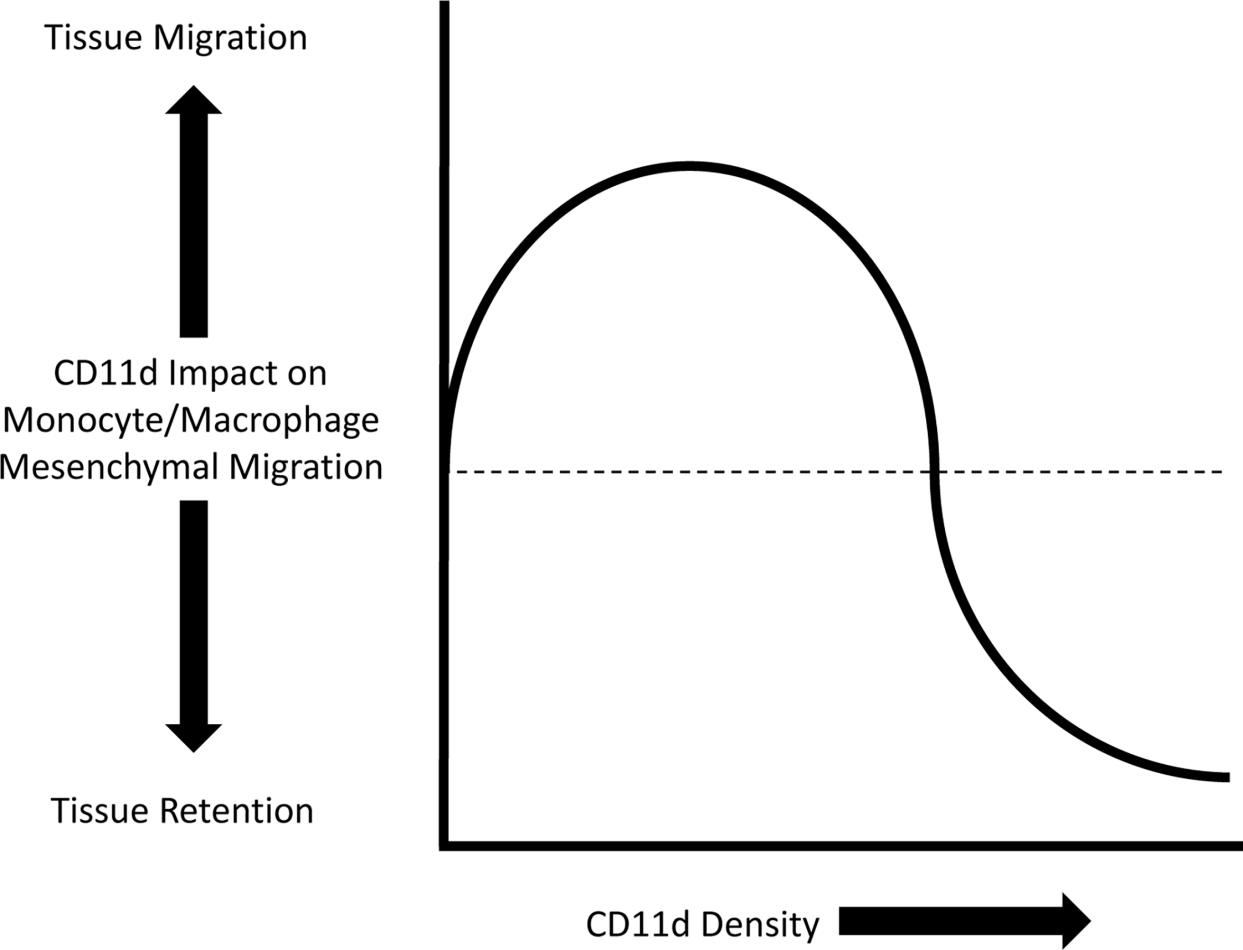
Representation of the impact CD11d density has on monocyte/macrophage mesenchymal migration. Low density of CD11d expression supports migration, while high densities inhibits migration and promotes tissue retention (8).

The modes of leukocyte tissue migration can mainly be divided into either amoeboid or mesenchymal migration. Amoeboid migration is characterized by weak adhesion to the ECM and the absence of ECM remodelling. In comparison, mesenchymal migration is characterized by integrin adhesion to the ECM and remodelling of the ECM by proteolysis (88). All leukocytes can employ amoeboid migration, while only monocytes/macrophages are able to partake in either amoeboid or mesenchymal migration (89). Variable densities of CD11d and CD11b differentially impact monocyte/macrophage mesenchymal migration (8, 90, 91). A mathematical model has previously described a bell curve relationship between cell adhesiveness and migration velocity (92). Integrin adhesion is dependent on integrin density, ligand affinity, and ligand density. An intermediate value of these three variables produces the maximum migration velocity (93). Low density of CD11d expression enhances mesenchymal migration, whereas high density arrests migration and promotes retention in inflamed tissue (8, 78) (**Figure 4**).

### Retention Following Lipid Peroxidation

The protein modification 2-(ω-carboxyethyl)-pyrrole (CEP) is a by-product of lipid peroxidation and a high affinity ligand for CD11d and CD11b (69). High affinity binding interactions between CEP adducts and integrins can increase the leukocyte adhesiveness and arrest monocyte/macrophage mesenchymal migration. During an inflammatory response, an increase in the abundance of CEP adducts can promote macrophage accumulation by arresting the migration of monocytes/macrophages (13, 69). Notably, integrin-CEP binding does not impact neutrophil migration, presumable because of their inability to participate in mesenchymal migration (69, 89). Neutrophils, however, can indirectly increase the prevalence of CEP adducts within the ECM upon activation and release of myeloperoxidase. Co-culture of myeloperoxidase and docosahexaenoate acid – a polyunsaturated fatty acid – increases the amount of CEP adducts in a fibrin matrix (69). These discoveries led to the model of a primary wave of neutrophils initiating lipid peroxidation and producing CEP adducts that “pave the way” for a secondary wave of macrophages (69). CEP adducts can arrest monocyte/macrophage migration because high affinity binding interactions result in substantial increases to leukocyte adhesiveness. CD11d is postulated to play the primary role in CEP adduct monocyte/macrophage retention because CD11d binds CEP adducts with an approximately 10-fold greater affinity than CD11b (69).

### M1/M2 Macrophage Migration

Macrophage heterogeneity heavily impacts the pro-inflammatory/anti-inflammatory balance within an inflammatory microenvironment (94, 95). The spectrum of macrophage heterogeneity can be described using a M1/M2 paradigm. M1 macrophages are pro-inflammatory pathogen-eliminating cells, whereas M2 macrophages are anti-inflammatory pro-wound healing cells. An immune response is typically organized by the primary infiltration of M1 macrophages before the secondary infiltration of M2 macrophages (94, 95). The cause of M1/M2 macrophage polarization is contentious and a detailed discussion can be found in the following review (94). Differential expression of CD11d has been demonstrated to form different migration patterns between M1 and M2 macrophages (78, 91). Strong expression of CD11d localizes M1 macrophages to sites of inflammation, while moderate CD11d expression on M2 macrophages is permissive for mesenchymal migration (91). These observations are supported by M2 macrophages participating in mesenchymal migration across a 3D matrix, while M1 macrophages are static (89).

Classical CD14^+^ monocytes are pro-inflammatory cells that primarily localize at the site of inflammation, while non-classical monocytes are pro-wound healing cells that primarily migrate and patrol (96). Under basal conditions both classical and non-classical monocytes express low levels of CD11d (7, 72). A contradiction exists between two studies, however, when reporting relative expression levels of CD11d between the monocyte sub-sets. One group reports that non-classical monocytes have the greater basal expression (72), while the second group reports greater expression amongst classical monocytes (7). Both groups analysed CD11d expression using flow cytometry. Resolving these reported contradictions will assist in determining if differential CD11d expression contributes to the staged migration patterns observed between monocyte sub-sets. In response to strenuous exercise, which does not alter CD11d expression, non-classical monocytes are mobilized, while classical monocytes were retained within the marginal pool (72). In models of neurotrauma (97) and myocardial infarction (98), classical monocytes are recruited to the site of inflammation several days before the secondary recruitment of non-classical monocytes. CD11d expression levels are increased in unfractionated monocytes following neurotrauma (74), but no direct link has been made between CD11d expression and these waves of monocyte sub-set recruitment. Investigation of the dynamic expression of CD11d amongst classical/non-classical monocytes and M1/M2 macrophages is warranted in determining if changes in CD11d levels can impact the staged migration of these cells. Furthering any knowledge on classical/non-classical monocyte or M1/M2 macrophage migration will be a valuable addition to the current discussion on the development of M1/M2 polarization during the progression of various pathophysiological states.

## Impact of CD11d Outside-In Signalling

β2 integrins have important outside-in signalling pathways induced by ligand binding. In general, integrins transduce outside-in signals from an active conformation; however, ligand binding to the inactive state can induce a conformational change and thereby transduce an outside-in signal (99). Antibody binding to the α-I domain in the presence of Mn^2+^ can also transmits outside-in signals as demonstrated in CD11b/CD18 (100). Canonical β2 integrin outside-in signalling pathways can impact cell motility, proliferation, survival, and cytokine expression. These detailed pathways are beyond the scope of this review and the reader can refer to the following reviews for in-depth analysis (2, 101). No outside-in signalling pathway has yet been elucidated for CD11d/CD18 but impacts of outside-in signalling have been described. Human monocytes incubated in wells coated with a variety of anti-CD11d murine monoclonal antibodies induced cell spreading along with the increased secretion of IL-8, IL-1β and MCP-1 (7). Human monocytes also secret IL-8 after binding human ICAM-3, but CD11d/CD18 is not the only integrin involved in binding ICAM-3 and transmitting an outside-in signal. Addition of a blocking anti-CD11d clone only partially reduces IL-8 secretion. CD11a/CD18 also recognizes ICAM-3 and could function redundantly to induce IL-8 secretion following ICAM-3 binding (7). A different signalling impact is detected for CD11d amongst human NK cells. Interactions between ICAM-3 on neutrophils and CD11d/CD18 on NK cells have been associated with IFN-γ release in co-cultures stimulated with LPS plus IL-15/IL-18 (73). Individually, NK cells also release IFN-γ following binding to immobilized ICAM-3 and IL-15/IL-18 stimulation. Application of a blocking anti-CD18 clone did abrogate NK cell IFN-γ release following ICAM-3 binding but could not differentiate between CD11d and CD11a signalling contributions (73). These studies highlight the presence of a CD11d/CD18 outside-in signalling cascade and the potential difficulties in separating the signalling contributions of individual integrins with shared ligand specificities. Future investigation is warranted to characterize the signalling molecules involved in the CD11d/CD18 outside-in signalling cascade. The distinct sequence variation in the CD11d cytoplasmic tail may indicate unique signalling pathways within the CD11d/CD18 outside-in signalling cascade not observed within the previously described canonical β2 integrin pathways.

## CD11d in Phagocytosis

β2 integrins as a family are known to participate in the phagocytosis of pathogens and senescent cells (102), but the role of CD11d in phagocytosis is largely undefined. The initial identification of CD11d expression on splenic red pulp macrophages linked their function with phagocytosis of spent and/or infected erythrocytes (5). In contrast to this original postulate, a study has demonstrated that CD11d/CD18 was not required for clearance of parasitized red blood cells in a murine malaria model (38). In CD11d*^-/-^* mice, peritoneal macrophages have no defect in the internalization of latex beads and phagocytosis of *Salmonella Typhimurium* (103). Additionally, a separate study found murine CD11d^-/-^ neutrophils and macrophages have no defect in the phagocytosis of *Escherichia Coli* (76). The current evidence, therefore, does not support CD11d/CD18 as a required participant in phagocytosis.

## CD11d in Macrophage Fusion

Both CD11b/CD18 and CD11d/CD18 have been associated with the formation of multinucleated giant cells (MNGCs) formed from the fusion of differentiated macrophages (104). The role an integrin plays in the process of macrophage fusion is thought to be proportional to the density of the integrin. CD11d/CD18 is expressed in a lower density than CD11b/CD18 on macrophages and likely plays a lesser role in macrophage fusion (104).

## CD11d Impact on T Cell Development

CD11d expression during thymocyte development impacts the immunological synapse and T cell proliferation (39). Thymocyte expression of both CD11b and CD11d has been reported to peak at days 12-17 in neonatal mice. Following thymic maturation, individual CD11b^-/-^ and CD11d^-/-^ knockout mice have the most severe T cell proliferation defects in response to staphylococcal enterotoxin (SE). The transient CD11b/CD11d co-expression is hypothesised to be crucial to T cell development as either knockout develops T cells with reduced CD3, CD28, CD4 and CD8 expression (39). Interestingly, CD11d^-/-^ mice display normal T cell proliferation in an experimental autoimmune encephalomyelitis (EAE) model (105). EAE is designed to model the autoimmune response to myelin oligodendrocyte glycoprotein (MOG) observed in multiple sclerosis (106). In this study susceptible CD11d^-/-^ mice were immunized with the MOG_35-55_ peptide to induce EAE (105). The MOG_35-55_ autoantigen induces a T cell MOG response, but no B cell MOG response (106). No difference in T cell proliferation was observed between susceptible CD11d^-/-^ and susceptible wildtype mice immunized with MOG_35-55_ (105). A separate EAE study noted an increase in CD11a-d/CD18 integrin expression amongst γδ T cells, while no change was observed amongst αβ T cells (75). Notably, CD11d is the only β2 integrin whose deletion does not improve EAE (105). Further work is warranted to investigate these conflicting T cell proliferation results and reveal the underlying mechanisms of CD11d/CD18 in thymocyte development.

## CD11d in Various Pathologies

### Atherosclerosis

Atherosclerosis is a chronic inflammatory disease of the cardiovascular system in which plaques narrow and harden arteries. Macrophage retention and foam cell formation at inflammatory sites along the arteries contribute to plaque lesion formation (46, 78, 107). Pro-inflammatory M1 macrophages dominate over anti-inflammatory M2 macrophages as the lesions and disease progress (107). The first connections between CD11d and atherosclerosis arose from observations of increased CD11d expression on foam cells in atherosclerotic lesions (5, 45). A recent CD11d knockout study found that CD11d^-/-^ mice had a decrease in atherosclerotic lesion severity, altered cytokine production, reduced lesion infiltration of M1 macrophages, and a decrease in macrophage CD36 expression (78). The CD11d^-/-^ mice had a decrease in Fas ligand, MIP-1α, IL-6, and IL-12 production, while an increase in IL-13 production was observed compared to wildtype. The observed change in M1 macrophage lesion accumulation was postulated to be caused by a change in macrophage mesenchymal migration. An increased CD11d density may promote macrophage retention instead of migration within the atherosclerotic lesion (78). Finally, CD36 acts as an Ox-LDL receptor that contributes to the accumulation of cytoplasmic Ox-LDL and macrophage foam cell differentiation. The connection between CD11d, CD36 signalling, and macrophage foam cell differentiation is currently under investigation (78).

### Obesity Driven Insulin Resistance

Insulin resistance caused by severe obesity is driven by chronic inflammation and macrophage infiltration into white adipose tissue (WAT) (108, 109). The progression to insulin resistance is characterized by the shift in WAT infiltrating M1 macrophages becoming predominant over M2 macrophages (109). Mouse models of obesity demonstrate an enormous 300-fold increase in CD11d mRNA levels within retroperitoneal WAT of obese animals compared to lean ones. A modest increase was also observed in CD11b, CD11c, VCAM-1, and ICAM-1 (81). The increase in β2 integrin expression is connected to macrophage infiltration by a correlated increase in the macrophage phagocytic marker CD68. Biopsies from female patients demonstrated a significant 6-fold increase in CD11d expression with subcutaneous WAT from obese patients compared to lean, but no trend was observed in omental WAT (81). Like the retention of M1 macrophages in atherosclerotic lesions, CD11d upregulation appears to drive M1 macrophage retention in WAT of obese patients. Targeting CD11d for reduced functional expression may imped the development of obesity-induced insulin resistance. CD11d^-/-^ mice have a reduced infiltration of macrophages into adipose tissue, improved glucose tolerance, and improved insulin sensitivity (91). A small molecule inhibitor of CD11d, P5 peptide, has been designed to bind to the CD11d α-I domain and block ligand binding interactions. In prediabetic mice, P5 peptide application was able to reduce the infiltration of adaptively transferred macrophages into WAT (13).

### Blood-Borne Pathogens

The strong expression of CD11d amongst splenic red pulp macrophages initiated the investigation into the role of CD11d in the clearance of blood-borne pathogens. Studies have shown that CD11d^-/-^ mice have increased survival in response to malarial *Plasmodium berghei* infection (38, 77), but a decreased survival in response to models of polymicrobial sepsis or *S. Typhimurium* infection (38, 76, 103). First, splenic red pulp macrophages are important mediators of parasitic red blood cell clearance and are maintained within the spleen in a specific microanatomic structure. CD11d^-/-^ mouse models of *P. berghei* infection demonstrate no defect in splenic microanatomy or parasitic clearance; however, systemic pro-inflammatory cytokines such as IL-12 were reduced compared to wildtype (38). The reduction in pro-inflammatory cytokines had a large impact on the development of malaria-associated acute respiratory distress syndrome (MA-ARDS) within the lungs. The lungs of CD11d^-/-^ mice had decreased levels of TNF, IL-1β, IL-6, IL-12, MCP-1, RANTES, and KC (a murine orthologue of IL-8). The reduction in pro-inflammatory cytokines was associated with decreased monocyte/macrophage lung infiltration, alveolar-capillary leakage, and mortality (77). In contrast, CD11d^-/-^ mice have an increased mortality following cecal ligation and puncture polymicrobial sepsis or LPS-induced endotoxemia (76). In response to LPS-induced endotoxemia, CD11d^-/-^ mice display a significant decrease in the number of monocytes/macrophages and an increase in the number of neutrophils that infiltrated into the lungs. No defect in phagocytosis is observed, but LPS treated CD11d^-/-^ neutrophils have a significant increase in necrosis and pyroptosis compared to wildtype neutrophils (76). The adaptive transfer of wildtype neutrophils, but not macrophages, is able to improve the survival of CD11d^-/-^ mice and is associated with an increase in the number of lung-infiltrating monocytes/macrophages. It is hypothesized that the increased macrophage number within the lungs was able to increase the effective efferocytosis of dead/necrotic neutrophils and confer the survival benefit (76). The protective role of CD11d in pyroptosis is supported by a peritoneal *S. Typhimurium* infection model. Increased pyrpotosis of peritoneal leukocytes in CD11d^-/-^ mice during *S. Typhimurium* infection is coupled with decreased pathogen killing and increased prevalence of pro-inflammatory cytokines TNFα, MIP-1α, and IL-6 compared to wildtype (103). Interestingly, the cytokine profiles of CD11d^-/-^ and wild type mice following LPS-induced endotoxemia are not significantly different (76). The perceived conflict described regarding the impact of CD11d on the survival to blood-borne infections may be resolved by the separate roles CD11d has on monocytes/macrophages and neutrophils. CD11d is known to impact monocyte/macrophage mesenchymal migration and monocyte CD11d outside-in signalling can release pro-inflammatory cytokines (7, 78, 110). In turn, neutrophils do not participate in mesenchymal migration that can be altered by CD11d density and little is known regarding neutrophil CD11d outside-in signalling (89). The protective mechanism of CD11d in neutrophil necrosis and pyroptosis is not clear, but LPS treatment does substantially increase neutrophil CD11d expression while not impacting macrophage CD11d expression (76). Neutrophils have been described as a “double-edge sword” during sepsis because the initial wave of neutrophils is key to combating the infection, but excessive neutrophil pyroptosis and release of pro-inflammatory mediators contributes to a harmful hyperinflammatory state (111). Therefore, the CD11d neutrophil mechanisms that are important protective factors in sepsis, but not parasitic infections, may resolve the discrepancy between CD11d expression and survival to various blood-borne pathogens.

### Neurotrauma

Neurotrauma is a complex injury that involves multiple injury stages that progress from an acute inflammatory state to a chronic inflammatory state. Following the primary injury, an influx of peripheral leukocytes into the CNS contributes to secondary damage through off-target effects (112). The progression of neurotrauma involves shifts in the prevalence of M1 *vs* M2 macrophages at the site of injury. Acute pro-inflammatory M1 macrophages predominate in the lesion within the acute 2-day period, while M2 macrophages predominate the subacute 7 to 14-day period. The subacute period is thought to aid wound healing and improve neurological recovery. A chronic inflammatory stage begins after day 14 and is characterized by the return to M1 macrophages (97). Unlike general trauma, neurotrauma induces an increase in CD11d and CD49d densities amongst neutrophils and monocytes expressing either of these integrins (74). Therapeutic antibodies have been designed against both CD11d and CD49d to prevent the acute extravasation and influx of peripheral leukocytes following neurotrauma (85, 113).

Administration of an anti-CD11d therapeutic at 2, 24, and 48 hours post-primary injury improves neurological recovery in rat and mouse models of spinal cord injury (9, 10) and rat models of traumatic brain injury (11, 12). Treatment is effective if provided within a 6-8 hour window following the primary injury and if the treatment continues for 48 hours (79, 114). First, the application of anti-CD11d reduces the acute infiltration of both neutrophils and monocyte/macrophages into the site of CNS injury (79, 85, 115). Changes to the leukocyte population within the lesion are coupled with dramatic changes to the inflammatory microenvironment. Compared to an isotype control, anti-CD11d induces a reduction in protein oxidation, DNA oxidation, lipid peroxidation, protein nitration, free radicals, and cell death (115–118). Microarray analysis elucidated substantial changes in gene expression following treatment, which peaked at day 3 post injury. Anti-CD11d treatment decreased the expression of IL-6 and IL-1β, while increasing CD4, CD8B, TLR4, and BMP7 (119). These changes to the inflammatory microenvironment are thought to be induced by the leukocyte population shift within the lesion but could also indicate alternative activation of leukocytes *via* the anti-CD11d treatment. Further investigation is warranted to determine if anti-CD11d treatment can induce outside-in signals and alternative activation of leukocytes. Regardless, the changes to the lesion microenvironment caused by anti-CD11d treatment spared myelin and improved the normality of white and grey matter architecture. Significant motor function improvements, reductions in mechanical allodynia, and reductions in autonomic dysreflexia were all observed following anti-CD11d treatment (9, 10, 120).

A key function of anti-CD11d treatment for acute neurotrauma is the temporal reduction of peripheral leukocyte infiltration into the site of CNS injury. CD11d can impact leukocyte localization by contributing to both extravasation and tissue migration. The inability of anti-CD11d treatment to improve the recovery of spinal cord injuries with substantial intraspinal haemorrhage indicates extravasation may be the main CD11d/CD18 mechanism driving peripheral leukocyte infiltration (86). Blocking CD49d/CD29 – an integrin that also contributes to VCAM-1-mediated extravasation – is also an effective strategy in treating acute neurotrauma (113). Together CD11d and CD49d may have a shared role in leukocyte extravasation or contribute to different stages of extravasation during leukocyte migration into the injury CNS. These therapeutics support the importance of extravasation of peripheral leukocytes during acute neurotrauma, especially in the setting of associated ischemia-reperfusion injury due to spinal cord compression.

Permitting the second wave of peripheral M2 macrophages is likely vital to the efficacy of anti-CD11d acute neurotrauma therapy. Methylprednisolone (MP), a previously standard of care for neurotrauma, is a general anti-inflammatory therapeutic that spares myelin but does not improve neurological recovery (121, 122). Both MP and anti-CD11d treatments reduce neutrophil and monocyte/macrophage infiltration within 3 days post-injury compared to untreated. Macrophage and neutrophil infiltration, however, is equal in anti-CD11d and untreated SCI lesions 7 days post-injury when the pro-wound healing M2 macrophages begin to predominate. In contrast, MP treatment decreased macrophage and increases neutrophil infiltration compared to untreated at day 7 post-injury (79). Combining both MP and anti-CD11d treatments abolishes the beneficial neurological improvement observed in anti-CD11d treatment alone (122). Modulating the waves of leukocyte infiltration, therefore, is more effective than blocking all waves of leukocyte infiltration into the CNS.

### Lung Injury

In response to lung infection or injury, an overreactive immune response can result in ARDS and oxygenation failure (123). The immunopathology of ARDS is exacerbated by the infiltration of peripheral monocytes and neutrophils. The infiltration of peripheral monocytes into the alveolar spaces increases pro-inflammatory mediators and furthers neutrophil recruitment (123, 124). Excessive neutrophil recruitment is harmful because netosis produces neutrophil extracellular traps that block the alveolar airway (123). Monocyte depletion in a mouse LPS-induced acute lung injury model can reduce neutrophil influx, TNF-α production, and the pathological score. It is hypothesized that the depletion of peripheral monocytes prevented the interactions between CD4^+^ T cells and monocytes that result in the differentiation of Th17 cells (124). Proliferation of Th17 cells in the lung is associated with an increase in the production of pro-inflammatory mediators that contribute to ARDS pathology, notably IL-17. Potential ARDS therapies, therefore, could target the migration of peripheral monocytes into the inflamed lungs (124).

Strong CD11d expression is observed in the lung tissue of deceased ARDS patients (7). A rat IgG-immune complex-induced lung injury model demonstrated CD11d upregulation within the lungs (80). Application of a rabbit polyclonal anti-CD11d, therefore, was hypothesized to decrease the severity of acute lung injury within the rat IgG-immune complex-induced model. Indeed, decreases in lung injury, neutrophil influx, TNF-α levels, and $NO_{2}^{-}/NO_{3}^{-}$ levels were observed following rabbit polyclonal anti-CD11d application. The production of $NO_{2}^{-}/NO_{3}^{-}$ in rat alveolar macrophages *in vitro* was also decreased by the application of the rabbit polyclonal anti-CD11d (80). Recalling the previous analysis of CD11d in MA-ARDS, decreased monocyte infiltration was observed in CD11d^-/-^ mice compared to wildtype. Furthermore, CD11d^-/-^ mice demonstrated reduced levels of pro-inflammatory cytokines including TNF and MCP-1 (77). In addition to lung infection or injury, ARDS can also be induced by systemic inflammation caused by trauma (123). Neurotrauma has been shown to induce systemic inflammatory response syndrome (SIRS), in which peripheral leukocytes infiltrate organs and cause damage (125–127). A retrospective study of 193 acute traumatic SCI patients found 47% had at least 2 SIRS criteria (128). Neurotrauma induced SIRS can induce life-threating lung damage and ARDS due to the infiltration of peripheral leukocytes (125). A single dose of anti-CD11d treatment at 2 hours following neurotrauma models can decrease neutrophil infiltration, macrophage accumulation, lipid peroxidation, protein nitration, and cell death within the lungs (126, 127). Anti-CD11d may abrogate the extravasation of peripheral monocytes and neutrophils into the alveolar spaces. Additionally, anti-CD11d may induce outside-in signalling that modulates the production of pro-inflammatory mediators by alveolar macrophages. Both CD11d/CD18 leukocyte localization and outside-signalling mechanisms, therefore, probably contribute to the immunopathology of acute lung injury and ARDS.

## CD11d-Targeted Therapies

Currently, two therapeutic agents have been developed to target CD11d and modulate leukocyte migration during disease/injury development. A therapeutic anti-CD11d monoclonal antibody has been developed to block the infiltration of peripheral leukocytes into the CNS during acute neurotrauma and extensively studied within *in vivo* mouse and rat models (9–11, 114, 115, 120). A peptide inhibitor, P5 peptide, has also been developed to block CD11d-ligand interactions during macrophage mesenchymal migration (13). *In vitro* P5 peptide experiments demonstrated that the therapeutic was able to alter wildtype macrophage migration in a 3D matrix, while not impacting diapedesis. The therapeutic was then able to successfully transition into an *in vivo* model of prediabetic mice to prevent the accumulation of macrophages in WAT (13). P5 peptide therapy, therefore, is positioned to treat chronic inflammatory diseases where CD11d contributes to the harmful accumulation of macrophages. Understanding off-target effects in addition to CD11d ligand binding are important in ensuring therapeutic safety. Both therapeutic agents target the ligand-binding α-I domain of CD11d, which could initiate integrin activation and/or outside-in signalling. Preliminary experiments report that P5 peptide may lock CD11d/CD18 into an intermediate state and prevent integrin activation (13). Further studies are still required to confirm this impact of P5 peptide on the CD11d/CD18 structure. Similar structural experiments between anti-CD11d therapy and CD11d/CD18 have yet to be performed. Previously, therapeutic attempts that targeted β2 integrins were not successful in translating into the clinic due to pervasive off-targeted effects. Efalizumab is an anti-CD11a therapeutic designed for long-term administration to treat chronic plaque psoriasis (129). CD11a is consistently expressed on all leukocytes and the systemic blockage of CD11a/CD18 activity led to severe side effects. Efalizumab was eventually discontinued due to the reactivation of JC virus and the development of fatal progressive multifocal leukoencephalopathy (130, 131). Currently, anti-CD11d therapeutics are mainly being developed for short-term use in acute inflammatory settings. A deeper understanding of CD11d expression and the affinity to each CD11d/CD18 conformation will assist in elucidating the range of potential side effects from these focused CD11d-targeted therapeutics.

## Conclusion

Integrins are an essential part of the immune system and the discovery of CD11d/CD18 expanded the breadth of the β2 integrin family. To date, however, CD11d/CD18 remains the least understood β2 integrin with major gaps in the knowledge of its structure and signalling pathways. Exciting discoveries have been made on the impact of CD11d/CD18 on leukocyte migration, retention, and coordination of a staged immune response. Emerging evidence demonstrates that differences in CD11d density may contribute to the differences in M1/M2 migration patterns, while CD11d specificity to CEP adducts from lipid peroxidation may contribute to the staging of neutrophil and monocyte/macrophage waves. CD11d-targeted therapeutic agents have been designed to modulate the localization of leukocytes during the progression of diseases or injuries. The dual impacts of CD11d/CD18 on cytokine release and localization of leukocytes, however, confound the therapeutic mechanism(s) of action that alter the inflammatory microenvironment. Ample opportunities exist to further the basic knowledge of CD11d/CD18 biology, which will propel the exciting developments of CD11d-targeted biological agents.

## Author Contributions

EB and GD contributed to the conception and outline of the review. EB wrote the first draft of the manuscript. GD, LW, and AB expanded sections of the manuscript. All authors contributed to manuscript revisions and have read and approved the submitted version.

## Funding

Funding that supported the creation of this manuscript was from the Canadian Institutes of Health grant OPG-363209 and by a donation from the National Hockey League Players Association.

## Conflict of Interest

The authors declare that the research was conducted in the absence of any commercial or financial relationships that could be construed as a potential conflict of interest.

## Publisher’s Note

All claims expressed in this article are solely those of the authors and do not necessarily represent those of their affiliated organizations, or those of the publisher, the editors and the reviewers. Any product that may be evaluated in this article, or claim that may be made by its manufacturer, is not guaranteed or endorsed by the publisher.

## References

[1] Fagerholm SC Guenther C Llort Asens M Savinko T Uotila LM. Beta2-Integrins and Interacting Proteins in Leukocyte Trafficking, Immune Suppression, and Immunodeficiency Disease. Front Immunol (2019) 10:254. doi: 10.3389/fimmu.2019.00254 30837997 PMC6389632

[2] Dib K. BETA 2 Integrin Signaling in Leukocytes. Front Biosci (2000) 5:438–51. doi: 10.2741/pathology 10762594

[3] Hu P Luo B-H. Integrin Bi-Directional Signaling Across the Plasma Membrane. J Cell Physiol (2013) 228:306–12. doi: 10.1002/jcp.24154 22767296

[4] Danilenko DM Rossitto PV Vieren MVd Trong HL McDonough SP Affolter VK. A Novel Canine Leukointegrin, Alpha D Beta 2, Is Expressed by Specific Macrophage Subpopulations in Tissue and a Minor CD8+ Lymphocyte Subpopulation in Peripheral Blood. J Immunol (1995) 155:35–44. 7541420

[5] Van der Vieren M Le Trong H Wood CL Moore PF St John T Staunton DE. A Novel Leukointegrin, Alpha D Beta 2, Binds Preferentially to ICAM-3. Immunity (1995) 3:683–90. doi: 10.1016/1074-7613(95)90058-6 8777714

[6] Siegers GM Barreira CR Postovit L-M Dekaban GA. CD11d β2 Integrin Expression on Human NK, B, and γδ T Cells. J Leukoc Biol (2017) 101:1029–35. doi: 10.1189/jlb.3AB0716-326RR 27881604

[7] Miyazaki Y Vieira-de-Abreu A Harris ES Shah AM Weyrich AS Castro-Faria-Neto HC. Integrin αdβ2 (CD11d/CD18) Is Expressed by Human Circulating and Tissue Myeloid Leukocytes and Mediates Inflammatory Signaling. PloS One (2014) 9:e112770. doi: 10.1371/journal.pone.0112770 25415295 PMC4240710

[8] Yakubenko VP Belevych N Mishchuk D Schurin A Lam SC-T Ugarova TP. The Role of Integrin αdβ2 (CD11d/CD18) in Monocyte/Macrophage Migration. Exp Cell Res (2008) 314:2569–78. doi: 10.1016/j.yexcr.2008.05.016 PMC2621015 18621369

[9] Gris D Marsh DR Oatway MA Chen Y Hamilton EF Dekaban GA. Transient Blockade of the CD11d/CD18 Integrin Reduces Secondary Damage After Spinal Cord Injury, Improving Sensory, Autonomic, and Motor Function. J Neurosci (2004) 24:4043–51. doi: 10.1523/JNEUROSCI.5343-03.2004 PMC6729422 15102919

[10] Geremia NM Bao F Rosenzweig TE Hryciw T Weaver L Dekaban GA. CD11d Antibody Treatment Improves Recovery in Spinal Cord-Injured Mice. J Neurotrauma (2012) 29:539–50. doi: 10.1089/neu.2011.1976 PMC4853192 22044160

[11] Utagawa A Bramlett HM Daniels L Lotocki G Dekaban G Weaver LC. Transient Blockage of the CD11d/CD18 Integrin Reduces Contusion Volume and Macrophage Infiltration After Traumatic Brain Injury in Rats. Brain Res (2008) 1207:155–63. doi: 10.1016/j.brainres.2008.02.057 PMC2435262 18374312

[12] Shultz SR Bao F Weaver LC Cain DP Brown A. Treatment With an Anti-CD11d Integrin Antibody Reduces Neuroinflammation and Improves Outcome in a Rat Model of Repeated Concussion. J Neuroinflammation (2013) 10:26. doi: 10.1186/1742-2094-10-26 23414334 PMC3598378

[13] Cui K Podolnikova NP Bailey W Szmuc E Podrez EA Byzova TV. Inhibition of Integrin αdβ2–Mediated Macrophage Adhesion to End Product of Docosahexaenoic Acid (DHA) Oxidation Prevents Macrophage Accumulation During Inflammation. J Biol Chem (2019) 294:14370–82. doi: 10.1074/jbc.RA119.009590 PMC6768641 31395659

[14] Campbell ID Humphries MJ. Integrin Structure, Activation, and Interactions. Cold Spring Harb Perspect Biol (2011) 3:a004994. doi: 10.1101/cshperspect.a004994 21421922 PMC3039929

[15] Evans R Patzak I Svensson L De Filippo K Jones K McDowall A. Integrins in Immunity. J Cell Sci (2009) 122:215–25. doi: 10.1242/jcs.019117 19118214

[16] Arnaout MA. Biology and Structure of Leukocyte β 2 Integrins and Their Role in Inflammation. F1000Res (2016) 5:F1000. doi: 10.12688/f1000research.9415.1 PMC5054827 27781085

[17] Xiong J-P Li R Essafi M Stehle T Arnaout MA. An Isoleucine-Based Allosteric Switch Controls Affinity and Shape Shifting in Integrin CD11b A-Domain*. J Biol Chem (2000) 275:38762–7. doi: 10.1074/jbc.C000563200 11034990

[18] Fan Z McArdle S Marki A Mikulski Z Gutierrez E Engelhardt B. Neutrophil Recruitment Limited by High-Affinity Bent β2 Integrin Binding Ligand in Cis. Nat Commun (2016) 7:12658. doi: 10.1038/ncomms12658 27578049 PMC5013657

[19] Fan Z Kiosses WB Sun H Orecchioni M Ghosheh Y Zajonc DM. High-Affinity Bent β2-Integrin Molecules in Arresting Neutrophils Face Each Other Through Binding to ICAMs In Cis. Cell Rep (2019) 26:119–130.e5. doi: 10.1016/j.celrep.2018.12.038 30605669 PMC6625519

[20] Saggu G Okubo K Chen Y Vattepu R Tsuboi N Rosetti F. Cis Interaction Between Sialylated Fcγriia and the αi-Domain of Mac-1 Limits Antibody-Mediated Neutrophil Recruitment. Nat Commun (2018) 9:5058. doi: 10.1038/s41467-018-07506-1 30498196 PMC6265255

[21] Sun Z Costell M Fässler R. Integrin Activation by Talin, Kindlin and Mechanical Forces. Nat Cell Biol (2019) 21:25–31. doi: 10.1038/s41556-018-0234-9 30602766

[22] Kadry YA Calderwood DA. Chapter 22: Structural and Signaling Functions of Integrins. Biochim Biophys Acta Biomembr (2020) 1862:183206. doi: 10.1016/j.bbamem.2020.183206 31991120 PMC7063833

[23] Patcha V Wigren J Winberg ME Rasmusson B Li J Särndahl E. Differential Inside-Out Activation of β2-Integrins by Leukotriene B4 and fMLP in Human Neutrophils. Exp Cell Res (2004) 300:308–19. doi: 10.1016/j.yexcr.2004.07.015 15474996

[24] Sampath R Gallagher PJ Pavalko FM. Cytoskeletal Interactions With the Leukocyte Integrin Beta2 Cytoplasmic Tail. Activation-Dependent Regulation of Associations With Talin and Alpha-Actinin. J Biol Chem (1998) 273:33588–94. doi: 10.1074/jbc.273.50.33588 PMC2823626 9837942

[25] Sen M Yuki K Springer TA. An Internal Ligand-Bound, Metastable State of a Leukocyte Integrin, αxβ2. J Cell Biol (2013) 203:629–42. doi: 10.1083/jcb.201308083 PMC3840939 24385486

[26] Gupta V Gylling A Alonso JL Sugimori T Ianakiev P Xiong J-P. The β-Tail Domain (βtd) Regulates Physiologic Ligand Binding to Integrin CD11b/Cd18. Blood (2007) 109:3513–20. doi: 10.1182/blood-2005-11-056689 PMC1852245 17170130

[27] Kechagia JZ Ivaska J Roca-Cusachs P. Integrins as Biomechanical Sensors of the Microenvironment. Nat Rev Mol Cell Biol (2019) 20:457–73. doi: 10.1038/s41580-019-0134-2 31182865

[28] ITGAM. (2004). Bethesda (MD): National Library of Medicine (US), National Center for Biotechnology Information. Available at: https://www.ncbi.nlm.nih.gov/gene/3684.

[29] ITGAX. (2004). Bethesda (MD): National Library of Medicine (US), National Center for Biotechnology Information. Available at: https://www.ncbi.nlm.nih.gov/gene/3687.

[30] ITGAD. (2004). Bethesda (MD): National Library of Medicine (US), National Center for Biotechnology Information. Available at: https://www.ncbi.nlm.nih.gov/gene/3681.

[31] ITGAL. (2004). Bethesda (MD): National Library of Medicine (US), National Center for Biotechnology Information. Available at: https://www.ncbi.nlm.nih.gov/gene/3683.

[32] Hughes AL. Evolution of the Integrin α and β Protein Families. J Mol Evol (2001) 52:63–72. doi: 10.1007/s002390010134 11139295

[33] Wong DA Davis EM LeBeau M Springer TA. Cloning and Chromosomal Localization of a Novel Gene-Encoding a Human Beta 2-Integrin Alpha Subunit. Gene (1996) 171:291–4. doi: 10.1016/0378-1119(95)00869-1 8666289

[34] Itgad. (2004). Bethesda (MD): National Library of Medicine (US), National Center for Biotechnology Information. Available at: https://www.ncbi.nlm.nih.gov/gene/381924.

[35] Itgax. (2004). Bethesda (MD): National Library of Medicine (US), National Center for Biotechnology Information. Available at: https://www.ncbi.nlm.nih.gov/gene/16411.

[36] Itgal. (2004). Bethesda (MD): National Library of Medicine (US), National Center for Biotechnology Information. Available at: https://www.ncbi.nlm.nih.gov/gene/16408.

[37] Itgam. (2004). Bethesda (MD): National Library of Medicine (US), National Center for Biotechnology Information. Available at: https://www.ncbi.nlm.nih.gov/gene/16409.

[38] Miyazaki Y Bunting M Stafforini DM Harris ES McIntyre TM Prescott SM. Integrin αdβ2 Is Dynamically Expressed by Inflamed Macrophages and Alters the Natural History of Lethal Systemic Infections. J Immunol (2008) 180:590–600. doi: 10.4049/jimmunol.180.1.590 18097061 PMC2275910

[39] Wu H Rodgers JR Perrard X-YD Perrard JL Prince JE Abe Y. Deficiency of CD11b or CD11d Results in Reduced Staphylococcal Enterotoxin-Induced T Cell Response and T Cell Phenotypic Changes. J Immunol (2004) 173:297–306. doi: 10.4049/jimmunol.173.1.297 15210787

[40] Noti JD Johnson AK Dillon JD. The Leukocyte Integrin Gene CD11d Is Repressed by Gut-Enriched Kruppel-Like Factor 4 in Myeloid Cells. J Biol Chem (2005) 280:3449–57. doi: 10.1074/jbc.M412627200 15561714

[41] Noti JD Johnson AK Dillon JD. The Zinc Finger Transcription Factor Transforming Growth Factor Beta-Inducible Early Gene-1 Confers Myeloid-Specific Activation of the Leukocyte Integrin CD11d Promoter. J Biol Chem (2004) 279:26948–58. doi: 10.1074/jbc.M310634200 15087465

[42] Noti JD Johnson AK Dillon JD. Structural and Functional Characterization of the Leukocyte Integrin Gene CD11d: ESSENTIAL ROLE OF Sp1 AND Sp3*. J Biol Chem (2000) 275:8959–69. doi: 10.1074/jbc.275.12.8959 10722744

[43] Okreglicka K Iten I Pohlmeier L Onder L Feng Q Kurrer M. Pparγ Is Essential for the Development of Bone Marrow Erythroblastic Island Macrophages and Splenic Red Pulp Macrophages. J Exp Med (2021) 218:e20191314. doi: 10.1084/jem.20191314 33765133 PMC8006858

[44] Standiford TJ Keshamouni VG Reddy RC. Peroxisome Proliferator-Activated Receptor-γ as a Regulator of Lung Inflammation and Repair. Proc Am Thorac Soc (2005) 2:226–31. doi: 10.1513/pats.200501-010AC 16222042

[45] Noti JD. Expression of the Myeloid-Specific Leukocyte Integrin Gene CD11d During Macrophage Foam Cell Differentiation and Exposure to Lipoproteins. Int J Mol Med (2002) 10:721–7. doi: 10.3892/ijmm.10.6.721 12429998

[46] Yu X-H Fu Y-C Zhang D-W Yin K Tang C-K. Foam Cells in Atherosclerosis. Clin Chim Acta (2013) 424:245–52. doi: 10.1016/j.cca.2013.06.006 23782937

[47] Fleming JC Norenberg MD Ramsay DA Dekaban GA Marcillo AE Saenz AD. The Cellular Inflammatory Response in Human Spinal Cords After Injury. Brain (2006) 129:3249–69. doi: 10.1093/brain/awl296 17071951

[48] Nagy L Tontonoz P Alvarez JG Chen H Evans RM. Oxidized LDL Regulates Macrophage Gene Expression Through Ligand Activation of PPARgamma. Cell (1998) 93:229–40. doi: 10.1016/s0092-8674(00)81574-3 9568715

[49] Taketa K Matsumura T Yano M Ishii N Senokuchi T Motoshima H. Oxidized Low Density Lipoprotein Activates Peroxisome Proliferator-Activated Receptor-Alpha (PPARalpha) and PPARgamma Through MAPK-Dependent COX-2 Expression in Macrophages. J Biol Chem (2008) 283:9852–62. doi: 10.1074/jbc.M703318200 18208815

[50] Park EJ Yuki Y Kiyono H Shimaoka M. Structural Basis of Blocking Integrin Activation and Deactivation for Anti-Inflammation. J BioMed Sci (2015) 22:51. doi: 10.1186/s12929-015-0159-6 26152212 PMC4495637

[51] Yakubenko VP Yadav SP Ugarova TP. Integrin αdβ2, an Adhesion Receptor Up-Regulated on Macrophage Foam Cells, Exhibits Multiligand-Binding Properties. Blood (2006) 107:1643–50. doi: 10.1182/blood-2005-06-2509 PMC1367263 16239428

[52] Hughes PE Diaz-Gonzalez F Leong L Wu C McDonald JA Shattil SJ. Breaking the Integrin Hinge: A Defined Structural Constraint Regulates Integrin Signaling. J Biol Chem (1996) 271:6571–4. doi: 10.1074/jbc.271.12.6571 8636068

[53] Jahan F Madhavan S Rolova T Viazmina L Grönholm M Gahmberg CG. Phosphorylation of the α-Chain in the Integrin LFA-1 Enables β2-Chain Phosphorylation and α-Actinin Binding Required for Cell Adhesion. J Biol Chem (2018) 293:12318–30. doi: 10.1074/jbc.RA118.004318 PMC6093247 29903913

[54] Fagerholm SC Varis M Stefanidakis M Hilden TJ Gahmberg CG. α-Chain Phosphorylation of the Human Leukocyte CD11b/CD18 (Mac-1) Integrin Is Pivotal for Integrin Activation to Bind ICAMs and Leukocyte Extravasation. Blood (2006) 108:3379–86. doi: 10.1182/blood-2006-03-013557 16857989

[55] Uotila LM Aatonen M Gahmberg CG. Integrin CD11c/CD18 α-Chain Phosphorylation Is Functionally Important. J Biol Chem (2013) 288:33494–9. doi: 10.1074/jbc.C113.497446 PMC3829194 24129562

[56] St-Denis N Gabriel M Turowec JP Gloor GB Li SS-C Gingras A-C. Systematic Investigation of Hierarchical Phosphorylation by Protein Kinase CK2. J Proteomics (2015) 118:49–62. doi: 10.1016/j.jprot.2014.10.020 25449829

[57] Thinn AMM Wang Z Zhu J. The Membrane-Distal Regions of Integrin α Cytoplasmic Domains Contribute Differently to Integrin Inside-Out Activation. Sci Rep (2018) 8:5067. doi: 10.1038/s41598-018-23444-w 29568062 PMC5864728

[58] Mahalingam B Ajroud K Alonso JL Anand S Adair B Horenstein AL. Stable Coordination of the Inhibitory Ca2+ Ion at MIDAS in Integrin CD11b/CD18 by an Antibody-Derived Ligand Aspartate: Implications for Integrin Regulation and Structure-Based Drug Design. J Immunol (2011) 187:6393–401. doi: 10.4049/jimmunol.1102394 PMC3237904 22095715

[59] Chatila TA Geha RS Arnaout MA. Constitutive and Stimulus-Induced Phosphorylation of CD11/CD18 Leukocyte Adhesion Molecules. J Cell Biol (1989) 109:3435–44. doi: 10.1083/jcb.109.6.3435 PMC2115914 2574726

[60] Buyon JP Slade SG Reibman J Abramson SB Philips MR Weissmann G. Constitutive and Induced Phosphorylation of the Alpha- and Beta-Chains of the CD11/CD18 Leukocyte Integrin Family. Relationship to Adhesion-Dependent Functions. J Immunol (1990) 144:191–7. 1967263

[61] Takala H Nurminen E Nurmi SM Aatonen M Strandin T Takatalo M. β2 Integrin Phosphorylation on Thr758 Acts as a Molecular Switch to Regulate 14-3-3 and Filamin Binding. Blood (2008) 112:1853–62. doi: 10.1182/blood-2007-12-127795 18550856

[62] Zhang XA Bontrager AL Stipp CS Kraeft S-K Bazzoni G Chen LB. Phosphorylation of a Conserved Integrin α3 QPSXXE Motif Regulates Signaling, Motility, and Cytoskeletal Engagement. Mol Biol Cell (2001) 12:351–65. doi: 10.1091/mbc.12.2.351 PMC30948 11179420

[63] Fagerholm SC Hilden TJ Gahmberg CG. P Marks the Spot: Site-Specific Integrin Phosphorylation Regulates Molecular Interactions. Trends Biochem Sci (2004) 29:504–12. doi: 10.1016/j.tibs.2004.07.005 15337124

[64] Qu A Leahy DJ. Crystal Structure of the I-Domain From the CD11a/CD18 (LFA-1, Alpha L Beta 2) Integrin. Proc Natl Acad Sci USA (1995) 92:10277–81. doi: 10.1073/pnas.92.22.10277 PMC40779 7479767

[65] Diamond MS Garcia-Aguilar J Bickford JK Corbi AL Springer TA. The I Domain Is a Major Recognition Site on the Leukocyte Integrin Mac-1 (CD11b/CD18) for Four Distinct Adhesion Ligands. J Cell Biol (1993) 120:1031–43. doi: 10.1083/jcb.120.4.1031 PMC2200080 7679388

[66] Bilsland CA Diamond MS Springer TA. The Leukocyte Integrin P150,95 (CD11c/CD18) as a Receptor for Ic3b. Activation by a Heterologous Beta Subunit and Localization of a Ligand Recognition Site to the I Domain. J Immunol (1994) 152:4582–9. 7512600

[67] Grayson MH van der Vieren M Sterbinsky SA Michael Gallatin W Hoffman PA Staunton DE. Alphadbeta2 Integrin Is Expressed on Human Eosinophils and Functions as an Alternative Ligand for Vascular Cell Adhesion Molecule 1 (VCAM-1). J Exp Med (1998) 188:2187–91. doi: 10.1084/jem.188.11.2187 PMC2212388 9841932

[68] Van der Vieren M Crowe DT Hoekstra D Vazeux R Hoffman PA Grayson MH. The Leukocyte Integrin Alpha D Beta 2 Binds VCAM-1: Evidence for a Binding Interface Between I Domain and VCAM-1. J Immunol (1999) 163:1984–90. 10438935

[69] Yakubenko VP Cui K Ardell CL Brown KE West XZ Gao D. Oxidative Modifications of Extracellular Matrix Promote the Second Wave of Inflammation *via* β2 Integrins. Blood (2018) 132:78–88. doi: 10.1182/blood-2017-10-810176 29724896 PMC6034644

[70] Baiula M Spampinato S Gentilucci L Tolomelli A. Novel Ligands Targeting α4β1 Integrin: Therapeutic Applications and Perspectives. Front Chem (2019) 7:489. doi: 10.3389/fchem.2019.00489 31338363 PMC6629825

[71] El-Gabalawy H Canvin J Ma GM Vieren Mvd Hoffman P Gallatin M. Synovial Distribution of αd/CD18, a Novel Leukointegrin. Comparison With Other Integrins and Their Ligands. Arthritis Rheum (1996) 39:1913–21. doi: 10.1002/art.1780391119 8912515

[72] Steppich B Dayyani F Gruber R Lorenz R Mack M Ziegler-Heitbrock HW. Selective Mobilization of CD14(+)CD16(+) Monocytes by Exercise. Am J Physiol Cell Physiol (2000) 279:C578–86. doi: 10.1152/ajpcell.2000.279.3.C578 10942707

[73] Costantini C Micheletti A Calzetti F Perbellini O Tamassia N Albanesi C. On the Potential Involvement of CD11d in Co-Stimulating the Production of Interferon-γ by Natural Killer Cells Upon Interaction With Neutrophils *via* Intercellular Adhesion Molecule-3. Haematologica (2011) 96:1543–7. doi: 10.3324/haematol.2011.044578 PMC3186317 21712539

[74] Bao F Bailey CS Gurr KR Bailey SI Rosas-Arellano MP Brown A. Human Spinal Cord Injury Causes Specific Increases in Surface Expression of β Integrins on Leukocytes. J Neurotrauma (2011) 28:269–80. doi: 10.1089/neu.2010.1618 PMC4851548 21142687

[75] Smith SS Barnum SR. Differential Expression of Beta 2-Integrins and Cytokine Production Between Gammadelta and Alphabeta T Cells in Experimental Autoimmune Encephalomyelitis. J Leukoc Biol (2008) 83:71–9. doi: 10.1189/jlb.0407263 17928460

[76] Bailey WP Cui K Ardell CL Keever KR Singh S Rodriguez-Gil DJ. Frontline Science: The Expression of Integrin αdβ2 (CD11d/CD18) on Neutrophils Orchestrates the Defense Mechanism Against Endotoxemia and Sepsis. J Leukoc Biol (2021) 109:877–90. doi: 10.1002/JLB.3HI0820-529RR PMC8085079 33438263

[77] de Azevedo-Quintanilha IG Vieira-de-Abreu A Ferreira AC Nascimento DO Siqueira AM Campbell RA. Integrin αdβ2 (CD11d/CD18) Mediates Experimental Malaria-Associated Acute Respiratory Distress Syndrome (MA-ARDS). Malar J (2016) 15:393. doi: 10.1186/s12936-016-1447-7 27473068 PMC4967320

[78] Aziz MH Cui K Das M Brown KE Ardell CL Febbraio M. The Upregulation of Integrin αdβ2 (CD11d/CD18) on Inflammatory Macrophages Promotes Macrophage Retention in Vascular Lesions and Development of Atherosclerosis. J Immunol (2017) 198:4855–67. doi: 10.4049/jimmunol.1602175 PMC5553324 28500072

[79] Saville LR Pospisil CH Mawhinney LA Bao F Simedrea FC Peters AA. A Monoclonal Antibody to CD11d Reduces the Inflammatory Infiltrate Into the Injured Spinal Cord: A Potential Neuroprotective Treatment. J Neuroimmunol (2004) 156:42–57. doi: 10.1016/j.jneuroim.2004.07.002 15465595

[80] Shanley TP Warner RL Crouch LD Dietsch GN Clark DL O’Brien MM. Requirements for Alpha D in IgG Immune Complex-Induced Rat Lung Injury. J Immunol (1998) 160:1014–20. 9551942

[81] Thomas AP Dunn TN Oort PJ Grino M Adams SH. Inflammatory Phenotyping Identifies CD11d as a Gene Markedly Induced in White Adipose Tissue in Obese Rodents and Women. J Nutr (2011) 141:1172–80. doi: 10.3945/jn.110.127068 21508205

[82] McKillop WM Barrett JW Pasternak SH Chan BMC Dekaban GA. The Extracellular Domain of CD11d Regulates Its Cell Surface Expression. J Leukoc Biol (2009) 86:851–62. doi: 10.1189/jlb.0309150 19571252

[83] Muller WA. Getting Leukocytes to the Site of Inflammation. Vet Pathol (2013) 50:7–22. doi: 10.1177/0300985812469883 23345459 PMC3628536

[84] Rutledge NS Muller WA. Understanding Molecules That Mediate Leukocyte Extravasation. Curr Pathobiol Rep (2020) 8:25–35. doi: 10.1007/s40139-020-00207-9

[85] Mabon PJ Weaver LC Dekaban GA. Inhibition of Monocyte/Macrophage Migration to a Spinal Cord Injury Site by an Antibody to the Integrin Alphad: A Potential New Anti-Inflammatory Treatment. Exp Neurol (2000) 166:52–64. doi: 10.1006/exnr.2000.7488 11031083

[86] Geremia NM Hryciw T Bao F Streijger F Okon E Lee JHT. The Effectiveness of the Anti-CD11d Treatment Is Reduced in Rat Models of Spinal Cord Injury That Produce Significant Levels of Intraspinal Hemorrhage. Exp Neurol (2017) 295:125–34. doi: 10.1016/j.expneurol.2017.06.002 28587875

[87] Küppers V Vestweber D Schulte D. Locking Endothelial Junctions Blocks Leukocyte Extravasation, But Not in All Tissues. Tissue Barriers (2013) 1:e23805. doi: 10.4161/tisb.23805 24665379 PMC3879176

[88] Friedl P Wolf K. Plasticity of Cell Migration: A Multiscale Tuning Model. J Cell Biol (2010) 188:11–9. doi: 10.1083/jcb.200909003 PMC2812848 19951899

[89] Cougoule C Van Goethem E Le Cabec V Lafouresse F Dupré L Mehraj V. Blood Leukocytes and Macrophages of Various Phenotypes Have Distinct Abilities to Form Podosomes and to Migrate in 3D Environments. Eur J Cell Biol (2012) 91:938–49. doi: 10.1016/j.ejcb.2012.07.002 22999511

[90] Lishko VK Yakubenko VP Ugarova TP. The Interplay Between Integrins Alphambeta2 and Alpha5beta1 During Cell Migration to Fibronectin. Exp Cell Res (2003) 283:116–26. doi: 10.1016/s0014-4827(02)00024-1 12565824

[91] Cui K Ardell CL Podolnikova NP Yakubenko VP. Distinct Migratory Properties of M1, M2, and Resident Macrophages Are Regulated by αdβ2 and αmβ2 Integrin-Mediated Adhesion. Front Immunol (2018) 9:2650. doi: 10.3389/fimmu.2018.02650 30524429 PMC6262406

[92] DiMilla PA Barbee K Lauffenburger DA. Mathematical Model for the Effects of Adhesion and Mechanics on Cell Migration Speed. Biophys J (1991) 60:15–37. doi: 10.1016/S0006-3495(91)82027-6 1883934 PMC1260035

[93] Palecek SP Loftus JC Ginsberg MH Lauffenburger DA Horwitz AF. Integrin-Ligand Binding Properties Govern Cell Migration Speed Through Cell-Substratum Adhesiveness. Nature (1997) 385:537–40. doi: 10.1038/385537a0 9020360

[94] Italiani P Boraschi D. From Monocytes to M1/M2 Macrophages: Phenotypical *vs.* Functional Differentiation. Front Immunol (2014) 5:514. doi: 10.3389/fimmu.2014.00514 25368618 PMC4201108

[95] Martinez FO Gordon S. The M1 and M2 Paradigm of Macrophage Activation: Time for Reassessment. F1000Prime Rep (2014) 6:13. doi: 10.12703/P6-13 24669294 PMC3944738

[96] Thomas G Tacke R Hedrick CC Hanna RN. Nonclassical Patrolling Monocyte Function in the Vasculature. Arterioscler Thromb Vasc Biol (2015) 35:1306–16. doi: 10.1161/ATVBAHA.114.304650 PMC4441550 25838429

[97] Thawer SG Mawhinney L Chadwick K de Chickera SN Weaver LC Brown A. Temporal Changes in Monocyte and Macrophage Subsets and Microglial Macrophages Following Spinal Cord Injury in the Lys-Egfp-Ki Mouse Model. J Neuroimmunol (2013) 261:7–20. doi: 10.1016/j.jneuroim.2013.04.008 23711349

[98] Nahrendorf M Swirski FK Aikawa E Stangenberg L Wurdinger T Figueiredo J-L. The Healing Myocardium Sequentially Mobilizes Two Monocyte Subsets With Divergent and Complementary Functions. J Exp Med (2007) 204:3037–47. doi: 10.1084/jem.20070885 PMC2118517 18025128

[99] Askari JA Buckley PA Mould AP Humphries MJ. Linking Integrin Conformation to Function. J Cell Sci (2009) 122:165–70. doi: 10.1242/jcs.018556 PMC2714414 19118208

[100] Lefort CT Hyun Y-M Schultz JB Law F-Y Waugh RE Knauf PA. Outside-In Signal Transmission by Conformational Changes in Integrin Mac-1. J Immunol (2009) 183:6460–8. doi: 10.4049/jimmunol.0900983 PMC2860599 19864611

[101] Schlaepfer DD Hunter T. Integrin Signalling and Tyrosine Phosphorylation: Just the FAKs? Trends Cell Biol (1998) 8:151–7. doi: 10.1016/s0962-8924(97)01172-0 9695829

[102] Sun H Zhi K Hu L Fan Z. The Activation and Regulation of β2 Integrins in Phagocytes and Phagocytosis. Front Immunol (2021) 12:633639. doi: 10.3389/fimmu.2021.633639 33868253 PMC8044391

[103] Nascimento D de O Vieira-de-Abreu A Arcanjo AF Bozza PT Zimmerman GA Castro-Faria-Neto HC. Integrin αdβ2 (CD11d/CD18) Modulates Leukocyte Accumulation, Pathogen Clearance, and Pyroptosis in Experimental Salmonella Typhimurium Infection. Front Immunol (2018) 9:1128. doi: 10.3389/fimmu.2018.01128 29881383 PMC5977906

[104] Podolnikova NP Kushchayeva YS Wu Y Faust J Ugarova TP. The Role of Integrins αmβ2 (Mac-1, CD11b/CD18) and αdβ2 (CD11d/CD18) in Macrophage Fusion. Am J Pathol (2016) 186:2105–16. doi: 10.1016/j.ajpath.2016.04.001 PMC4973655 27315778

[105] Adams JE Webb MS Hu X Staunton D Barnum SR. Disruption of the β2-Integrin CD11d (αdβ2) Gene Fails to Protect Against Experimental Autoimmune Encephalomyelitis. J Neuroimmunol (2007) 184:180–7. doi: 10.1016/j.jneuroim.2006.12.007 PMC2747331 17254640

[106] Dang AK Jain RW Craig HC Kerfoot SM. B Cell Recognition of Myelin Oligodendrocyte Glycoprotein Autoantigen Depends on Immunization With Protein Rather Than Short Peptide, While B Cell Invasion of the CNS in Autoimmunity Does Not. J Neuroimmunol (2015) 278:73–84. doi: 10.1016/j.jneuroim.2014.12.008 25595255

[107] Khallou-Laschet J Varthaman A Fornasa G Compain C Gaston A-T Clement M. Macrophage Plasticity in Experimental Atherosclerosis. PloS One (2010) 5:e8852. doi: 10.1371/journal.pone.0008852 20111605 PMC2810335

[108] Weisberg SP McCann D Desai M Rosenbaum M Leibel RL Ferrante AW. Obesity Is Associated With Macrophage Accumulation in Adipose Tissue. J Clin Invest (2003) 112:1796–808. doi: 10.1172/JCI19246 PMC296995 14679176

[109] Olefsky JM Glass CK. Macrophages, Inflammation, and Insulin Resistance. Annu Rev Physiol (2010) 72:219–46. doi: 10.1146/annurev-physiol-021909-135846 20148674

[110] Yakubenko VP Belevych N Mishchuk D Schurin A Lam SC-T Ugarova TP. The Role of Integrin αdβ2 (CD11d/CD18) in Monocyte/Macrophage Migration. Exp Cell Res (2008) 314:2569–78. doi: 10.1016/j.yexcr.2008.05.016 PMC2621015 18621369

[111] Liu L Sun B. Neutrophil Pyroptosis: New Perspectives on Sepsis. Cell Mol Life Sci (2019) 76:2031–42. doi: 10.1007/s00018-019-03060-1 PMC11105444 30877336

[112] Mietto BS Mostacada K Martinez AMB. Neurotrauma and Inflammation: CNS and PNS Responses. Mediators Inflamm (2015) 2015:251204. doi: 10.1155/2015/251204 25918475 PMC4397002

[113] Fleming JC Bao F Chen Y Hamilton EF Relton JK Weaver LC. Alpha4beta1 Integrin Blockade After Spinal Cord Injury Decreases Damage and Improves Neurological Function. Exp Neurol (2008) 214:147–59. doi: 10.1016/j.expneurol.2008.04.024 19038604

[114] Ditor DS Bao F Chen Y Dekaban GA Weaver LC. A Therapeutic Time Window for Anti-CD11d Monoclonal Antibody Treatment Yielding Reduced Secondary Tissue Damage and Enhanced Behavioral Recovery Following Severe Spinal Cord Injury. J Neurosurg Spine (2006) 5:343–52. doi: 10.3171/spi.2006.5.4.343 17048772

[115] Bao F Shultz SR Hepburn JD Omana V Weaver LC Cain DP. A CD11d Monoclonal Antibody Treatment Reduces Tissue Injury and Improves Neurological Outcome After Fluid Percussion Brain Injury in Rats. J Neurotrauma (2012) 29:2375–92. doi: 10.1089/neu.2012.2408 PMC4854615 22676851

[116] Bao F Chen Y Dekaban GA Weaver LC. An Anti-CD11d Integrin Antibody Reduces Cyclooxygenase-2 Expression and Protein and DNA Oxidation After Spinal Cord Injury in Rats. J Neurochem (2004) 90:1194–204. doi: 10.1111/j.1471-4159.2004.02580.x 15312174

[117] Bao F Chen Y Dekaban GA Weaver LC. Early Anti-Inflammatory Treatment Reduces Lipid Peroxidation and Protein Nitration After Spinal Cord Injury in Rats. J Neurochem (2004) 88:1335–44. doi: 10.1046/j.1471-4159.2003.02240.x 15009633

[118] Bao F Dekaban GA Weaver LC. Anti-CD11d Antibody Treatment Reduces Free Radical Formation and Cell Death in the Injured Spinal Cord of Rats. J Neurochem (2005) 94:1361–73. doi: 10.1111/j.1471-4159.2005.03280.x 15992367

[119] Gris P Tighe A Thawer S Hemphill A Oatway M Weaver L. Gene Expression Profiling in Anti-CD11d mAb-Treated Spinal Cord-Injured Rats. J Neuroimmunol (2009) 209:104–13. doi: 10.1016/j.jneuroim.2009.02.002 19250688

[120] Oatway MA Chen Y Bruce JC Dekaban GA Weaver LC. Anti-CD11d Integrin Antibody Treatment Restores Normal Serotonergic Projections to the Dorsal, Intermediate, and Ventral Horns of the Injured Spinal Cord. J Neurosci (2005) 25:637–47. doi: 10.1523/JNEUROSCI.3960-04.2005 PMC6725335 15659600

[121] Gris D Marsh DR Dekaban GA Weaver LC. Comparison of Effects of Methylprednisolone and Anti-CD11d Antibody Treatments on Autonomic Dysreflexia After Spinal Cord Injury. Exp Neurol (2005) 194:541–9. doi: 10.1016/j.expneurol.2005.03.016 15890340

[122] Weaver LC Gris D Saville LR Oatway MA Chen Y Marsh DR. Methylprednisolone Causes Minimal Improvement After Spinal Cord Injury in Rats, Contrasting With Benefits of an Anti-Integrin Treatment. J Neurotrauma (2005) 22:1375–87. doi: 10.1089/neu.2005.22.1375 16379576

[123] Wong JJM Leong JY Lee JH Albani S Yeo JG. Insights Into the Immuno-Pathogenesis of Acute Respiratory Distress Syndrome. Ann Transl Med (2019) 7:504. doi: 10.21037/atm.2019.09.28 31728357 PMC6828790

[124] Jiang Z Zhou Q Gu C Li D Zhu L. Depletion of Circulating Monocytes Suppresses IL-17 and HMGB1 Expression in Mice With LPS-Induced Acute Lung Injury. Am J Physiol Lung Cell Mol Physiol (2017) 312:L231–42. doi: 10.1152/ajplung.00389.2016 27913426

[125] Gris D Hamilton EF Weaver LC. The Systemic Inflammatory Response After Spinal Cord Injury Damages Lungs and Kidneys. Exp Neurol (2008) 211:259–70. doi: 10.1016/j.expneurol.2008.01.033 18384773

[126] Weaver LC Bao F Dekaban GA Hryciw T Shultz SR Cain DP. CD11d Integrin Blockade Reduces the Systemic Inflammatory Response Syndrome After Traumatic Brain Injury in Rats. Exp Neurol (2015) 271:409–22. doi: 10.1016/j.expneurol.2015.07.003 PMC4854624 26169930

[127] Bao F Brown A Dekaban GA Omana V Weaver LC. CD11d Integrin Blockade Reduces the Systemic Inflammatory Response Syndrome After Spinal Cord Injury. Exp Neurol (2011) 231:272–83. doi: 10.1016/j.expneurol.2011.07.001 PMC4853193 21784069

[128] Kesani AK Urquhart JC Bedard N Leelapattana P Siddiqi F Gurr KR. Systemic Inflammatory Response Syndrome in Patients With Spinal Cord Injury: Does Its Presence at Admission Affect Patient Outcomes?: Clinical Article. J Neurosurg Spine (2014) 21:296–302. doi: 10.3171/2014.3.SPINE13784 24836657

[129] Leonardi CL Papp KA Gordon KB Menter A Feldman SR Caro I. Efalizumab Study Group. Extended Efalizumab Therapy Improves Chronic Plaque Psoriasis: Results From a Randomized Phase III Trial. J Am Acad Dermatol (2005) 52:425–33. doi: 10.1016/j.jaad.2004.09.029 15761420

[130] Schwab N Ulzheimer JC Fox RJ Schneider-Hohendorf T Kieseier BC Monoranu CM. Fatal PML Associated With Efalizumab Therapy: Insights Into Integrin αlβ2 in JC Virus Control. Neurology (2012) 78:458–67. doi: 10.1212/WNL.0b013e3182478d4b PMC3280052 22302546

[131] Schittenhelm L Hilkens CM Morrison VL. β2 Integrins As Regulators of Dendritic Cell, Monocyte, and Macrophage Function. Front Immunol (2017) 8:1866. doi: 10.3389/fimmu.2017.01866 29326724 PMC5742326

